# Supplementary material for: From structural polymorphism to structural metamorphosis of the coat protein of flexuous filamentous potato virus Y
Source: Commun Chem. 2024 Jan 17;7:14. doi: 10.1038/s42004-024-01100-x (PMC10794713; doi:10.1038/s42004-024-01100-x)
Supplement: Supplementary file 2 — Supplementary Information [file 42004_2024_1100_MOESM2_ESM.pdf]

Supplementary Information for

From structural polymorphism to structural metamorphosis of the coat protein of flexuous filamentous potato virus Y

Luka Kavčič, Andreja Kežar, Neža Koritnik, Magda Tušek Žnidarič, Tajda Klobučar, Žiga Vičič, Franci Merzel, Ellie Holden, Justin L. P. Benesch and Marjetka Podobnik

Correspondence to: marjetka.podobnik@ki.si

The PDF file includes:

Supplementary Note 1
Supplementary Figs 1 to 25
Supplementary Tables 1 to 2
Supplementary References

Supplementary Note 1

Each coding sequence (CDS) in pRSF-Duet1 is preceded by one T7 promotor and there is only one T7 terminator after the second MCS. *CP* was inserted in MCS1 and *P97* in MCS2 (Fig. 6f), which facilitated production of two mRNA transcripts, one encoding both CP and p97 (*CP-P97*) and the other only p97 (*p97*). However, RNA sequencing analysis revealed uneven coverage along both CDS for total cell RNA sample, with a peculiarly sharp drop in coverage near position 1500 along the combined transcript (vertical dotted line on Fig. 8g), where one would expect constant coverage or at least higher than the values along CP CDS due to the production of both *CP-p97* and *p97* mRNA. Presence of p97 protein in cell lysates showed that *p97* mRNA was successfully produced a prerequisite for production of full-length p97 protein able to form characteristic hexamers (Supplementary Fig. 25a), and that *CP-P97* transcript was likely truncated and therefore it was consequently marked as *CP-P97**. While we have no direct explanation why this truncation occurred only in *CP-p97*, it could result from the premature transcription termination of *CP-P97** transcript due to the presence of iTerm-PseKNC[1]-predicted Rho-independent intrinsic terminator sites in the region after position 1500. Nevertheless, the low coverage of the region after 1500 in analysis of the RNA from VLPs (Fig. 8g) clearly shows that while *CP-P97** is efficiently encapsidated inside VLPs, the full-length *P97* is not.

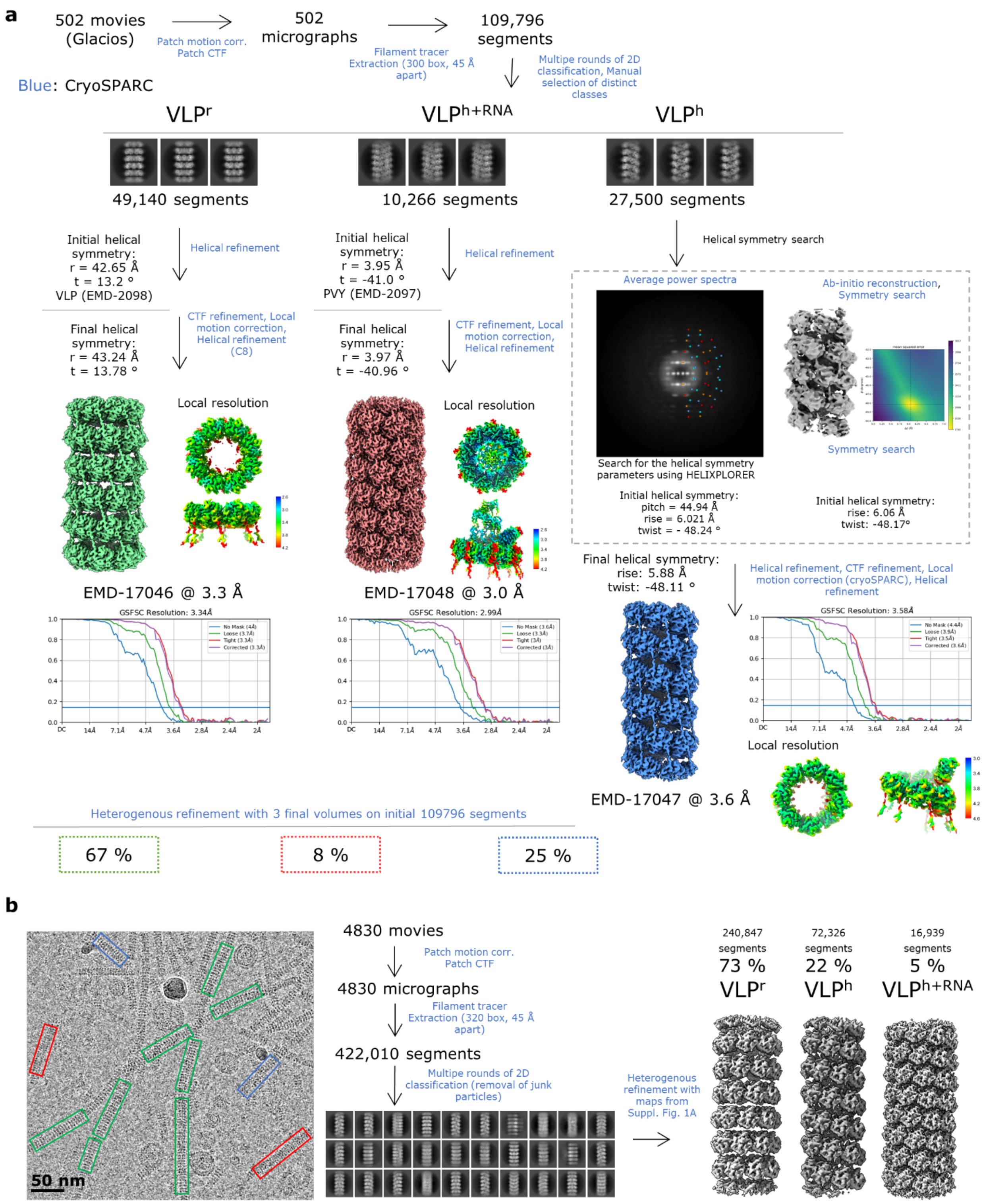

Supplementary Figure 1: Cryo-EM data processing of VLP.

a, Workflow of cryo-EM data processing for wild type VLPs prepared in this study. ‘r’ and ‘t’ denote the experimentally determined rise and twist values of helical symmetry, respectively. **b,** Workflow of reanalysis of cryo-EM data for wild type VLPs from our previous study[2]. The particles on the micrograph (left) are marked based on the morphology: VLP^{r}: green, VLP^{h}: blue, VLP^{h+RNA}: red.

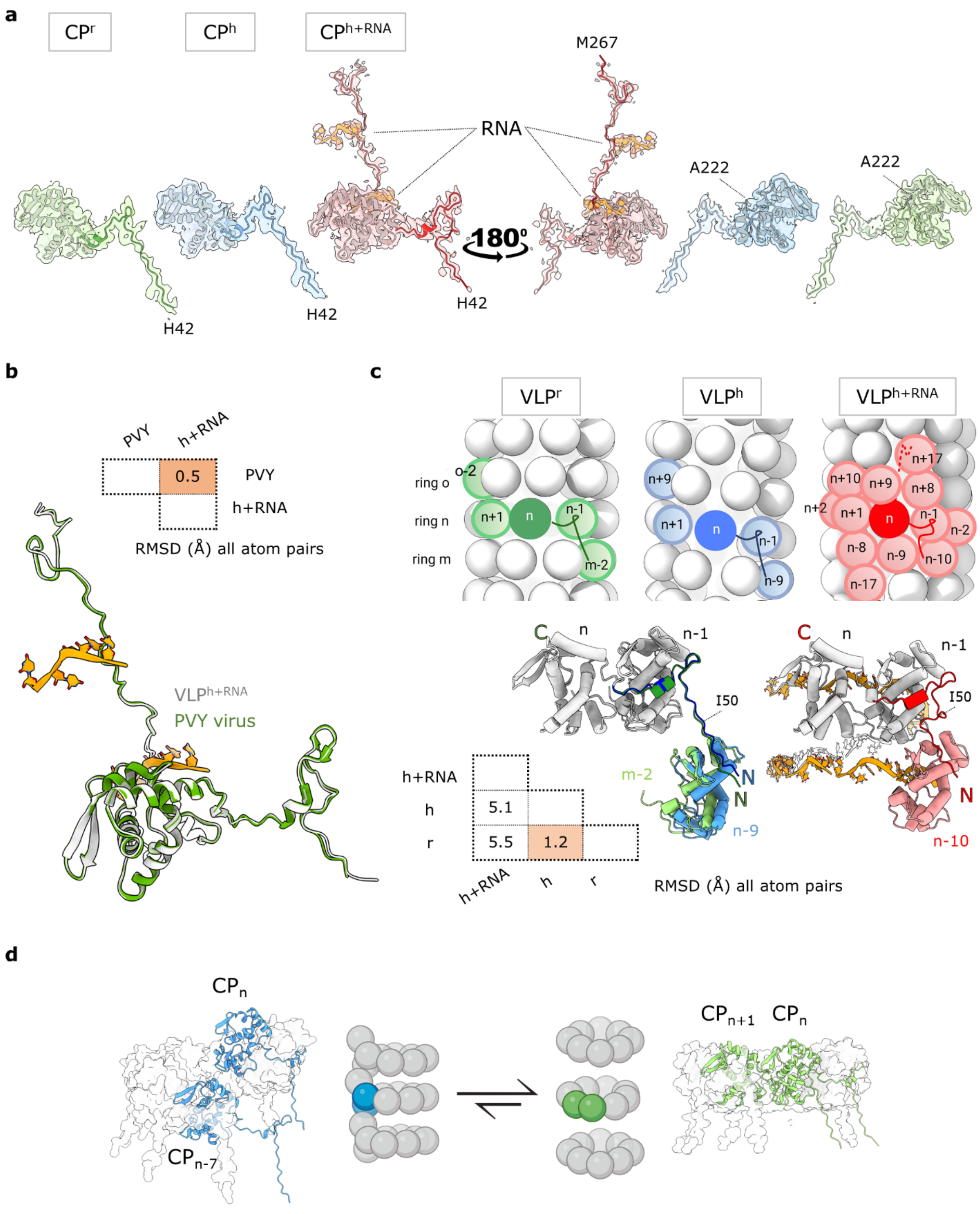

Supplementary Figure 2: Structural analysis of wild type VLPs.

a, Fit of atomic models of CP subunits into cryo-EM density maps of VLP^{r}, VLP^{h} and VLP^{h+RNA} with first and last determined amino acid residue marked. **b,** Superposition of all atoms in CP^{h+RNA} from VLP^{h+RNA} (white) and CP unit from PVY virion (PDB-ID 6HXX) (green) with RNA in orange cartoon. **c,** Top:

schematic representation of the interacting CP subunits in the three VLP forms. The core region of the CP unit 'n'(CP_n) is drawn as a full circle and the surface-exposed N-IDR interacting with other CP subunits as a line. In VLP^{h+RNA}, the luminal C-IDR is shown as a dashed red line and is not depicted in VLP^r and VLP^h because it is disordered. Bottom left: Superposition of three CP units as labelled above, connected by the N-IDR, as determined in VLP^r (green) and VLP^h (blue). In this case, the CP core regions (N76-A222) of the subunit (CP_{n-1}) were aligned (as in Fig. 1d), with RMSD values between all atoms provided in Å. For comparison (bottom right), three linked subunits in VLP^{h+RNA} (gray/red) are shown, with RNA in orange. **d,** the scheme depicts potential equilibrium between the helically arranged CP^h protomers (blue) and the CP^r octameric ring (green), resulting in different RNA-free filament architectures.

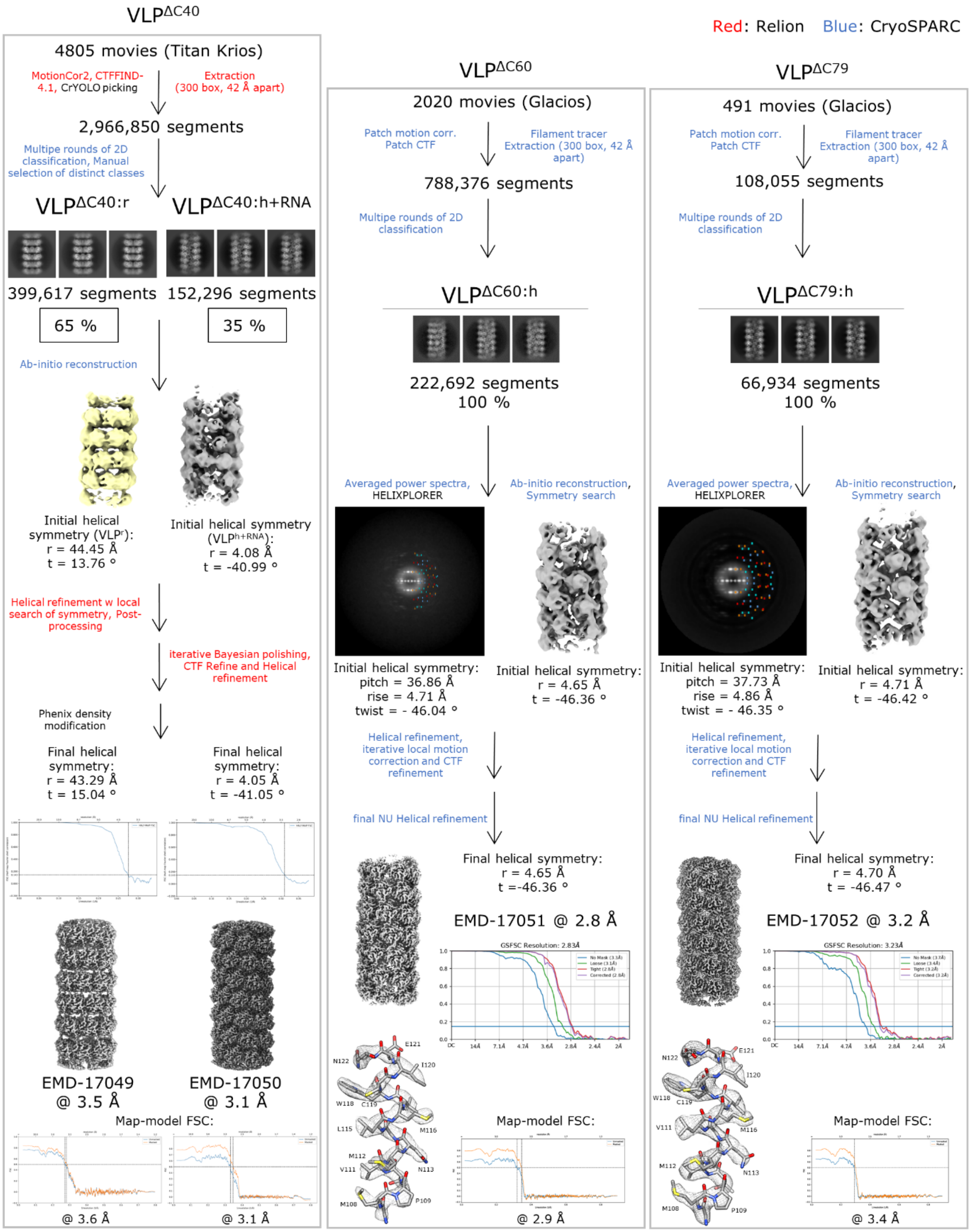

Supplementary Figure 3: Cryo-EM data processing for VLP$^{\Delta C40}$, VLP$^{\Delta C60}$, and VLP$^{\Delta C79}$.
Cryo-EM data processing workflow for VLP$^{\Delta C40}$ (left), VLP$^{\Delta C60}$ (middle), and VLP$^{\Delta C79}$ (right).

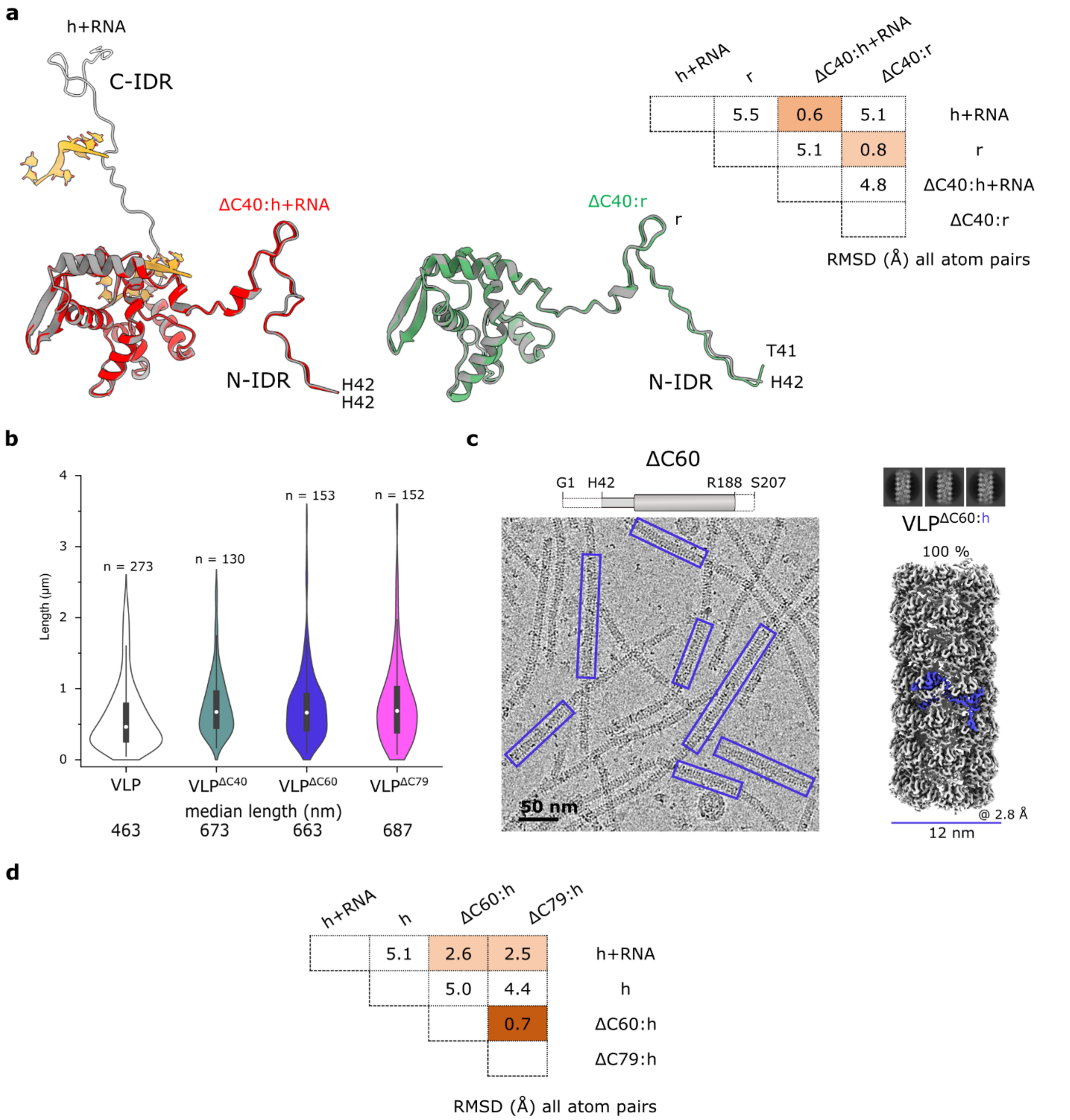

Supplementary Figure 4: Structural analysis of filaments formed by CP truncated at the C-terminus. **a,** Left: superposition of $CP^{\Delta C40:h+RNA}$ (red) with wild type CP^{h+RNA}. Right: superposition of $CP^{\Delta C40:r}$ (green) and wild type CP^{r} (gray). Alignment was performed on the core regions (N76-A222), with RMSD values between all atoms provided in Å. **b,** Violin plot of filament length distribution for wild type VLP (white), $VLP^{\Delta C40}$ (green), $VLP^{\Delta C60}$ (blue), and $VLP^{\Delta C79}$ (pink) with median provided below. The number of measured filaments (‘n’) is shown above each violin. The median value and the area above the 25th and below the 75th percentile are indicated with a white circle and a black filled rectangle, respectively. All filament length measurements are provided in the Supplementary Data file. **c,** Top left: schematic representation of the $CP^{\Delta C60}$ construct with dashed regions G1-T41 and N199-S207 not defined in the cryo-

EM density map. Bottom left: representative VLP$^{\Delta C60}$ cryo-EM micrograph showing mapped filaments of a single type (blue rectangles). Top right: 2D class averages of VLP$^{\Delta C60:h}$ particles. Bottom right: 3D reconstruction of VLP$^{\Delta C60:h}$ with filament diameter in nm and overall resolution in Å. **d,** RMSD values for all atom pairs in Å for the superposition on Fig. 1g, the alignment was performed on the residues of the core region (N76-A222).

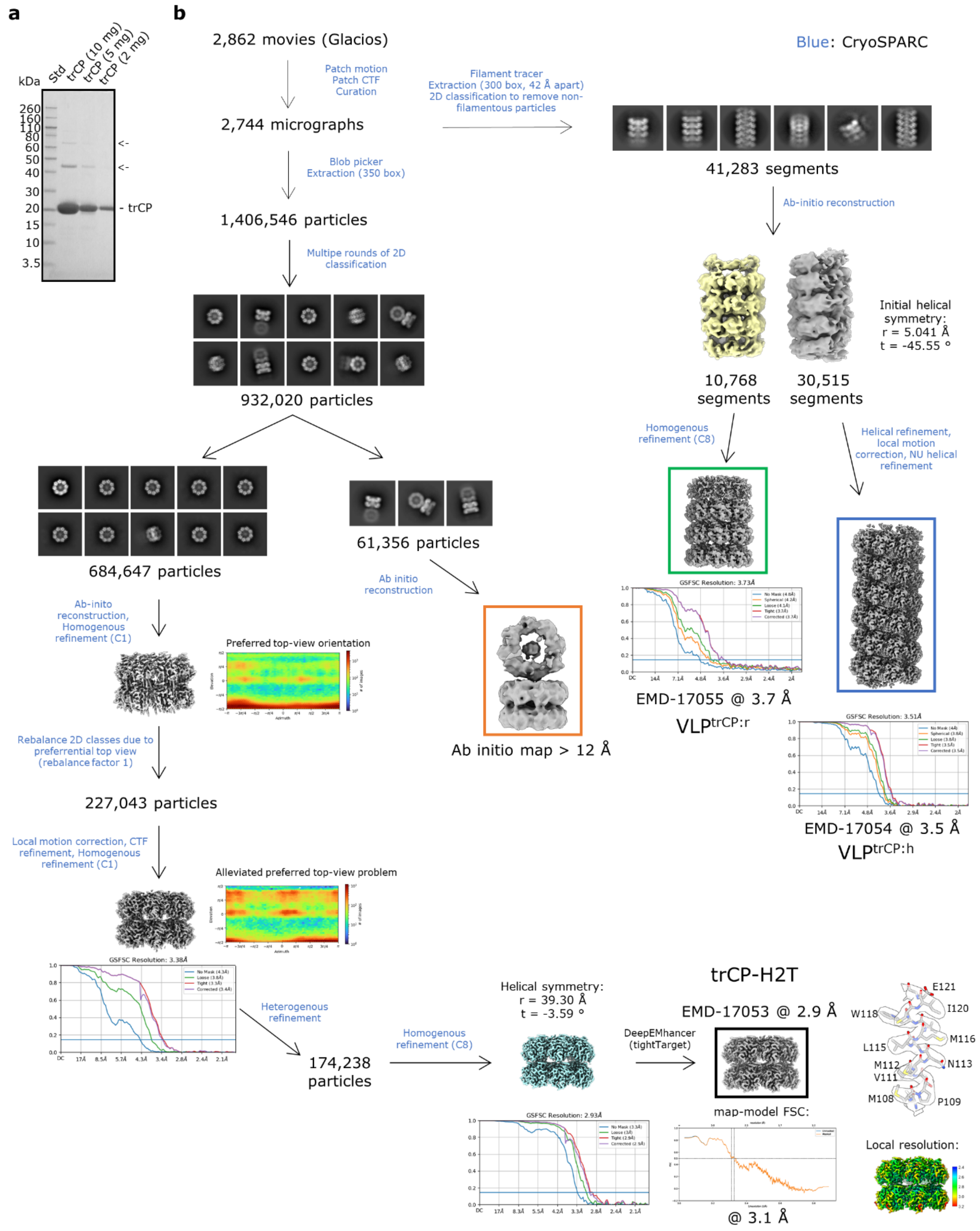

Supplementary Figure 5: Cryo-EM data processing of particles formed by trCP.

a, SDS PAGE analysis of trCP sample. The band for trCP monomer is marked (- trCP) and the bands representing the trCP oligomers with arrows. Std: molecular weight standards. The source data is provided as Supplementary Data file. **b,** Workflow of cryo-EM data processing for trCP assemblies from Fig. 2a.

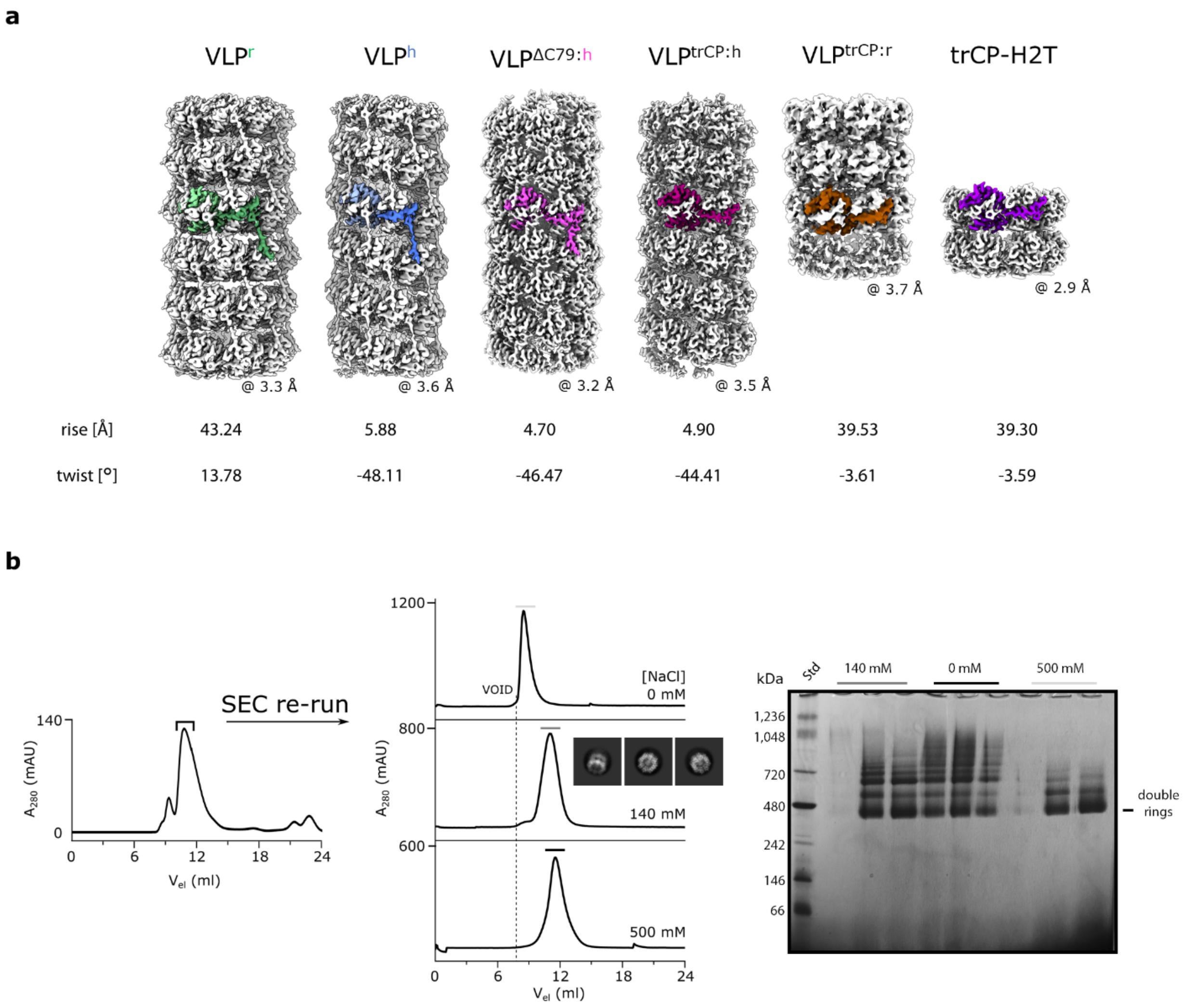

Supplementary Figure 6: Structural comparison of trCP self-assemblies with VLP^{h}, VLP^{r}, and VLP$^{\Delta C79:h}$ and propensity to associate despite truncated IDRs.

a, Comparison of cryo-EM 3D reconstructions of wild type VLP^{r} (green) and VLP^{h} (blue), VLP$^{\Delta C79:h}$ (pink), trCP helical (VLP$^{trCP:h}$, purple) and stacked ring filaments (VLP$^{trCP:r}$, brown), and trCP H2T double-rings (trCP-H2T, magenta) with the corresponding helical parameters below. **b,** Left: SEC analysis (Superdex200 10/300 GL) of the trCP sample. The main fraction (marked) was run again on SEC using different NaCl concentration (center). The major fractions from these SEC analyses were further analyzed on native-PAGE (right) Std: molecular weight standards. The source data are provided as Supplementary Data file.

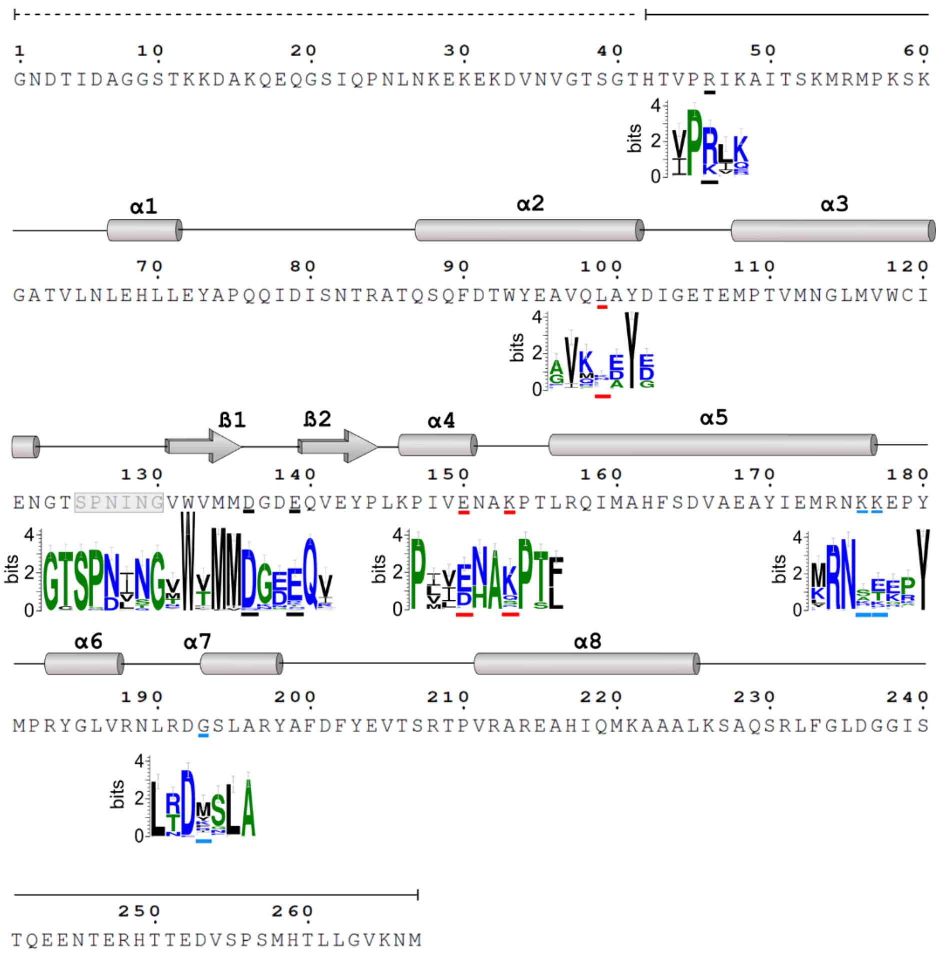

Supplementary Figure 7: Schematic representation of CP amino acid sequence and secondary structural elements in CP^{h+RNA}.

Schematic representation of the CP amino acid sequence (translated from GenBank no. KM396648, PVY^{NTN} isolate). RNA-binding loop S125-G130 is highlighted with light gray, residues from the charged triangle R46/D136/E139 are underlined in black, non-conserved residues from the 'N-side' in red and from the 'P-side' in blue (see Fig. 2d). Sequence logos indicating sequence conservation were generated using WebLogo3 based on the result of Clustal Omega multiple sequence alignment of 64 different representatives of the potyvirus genus[3]. Elements of secondary structure as determined in CP^{h+RNA} are drawn above the amino acid sequence with dotted line indicating first 41 residues that could not be modeled in the cryo-EM map.

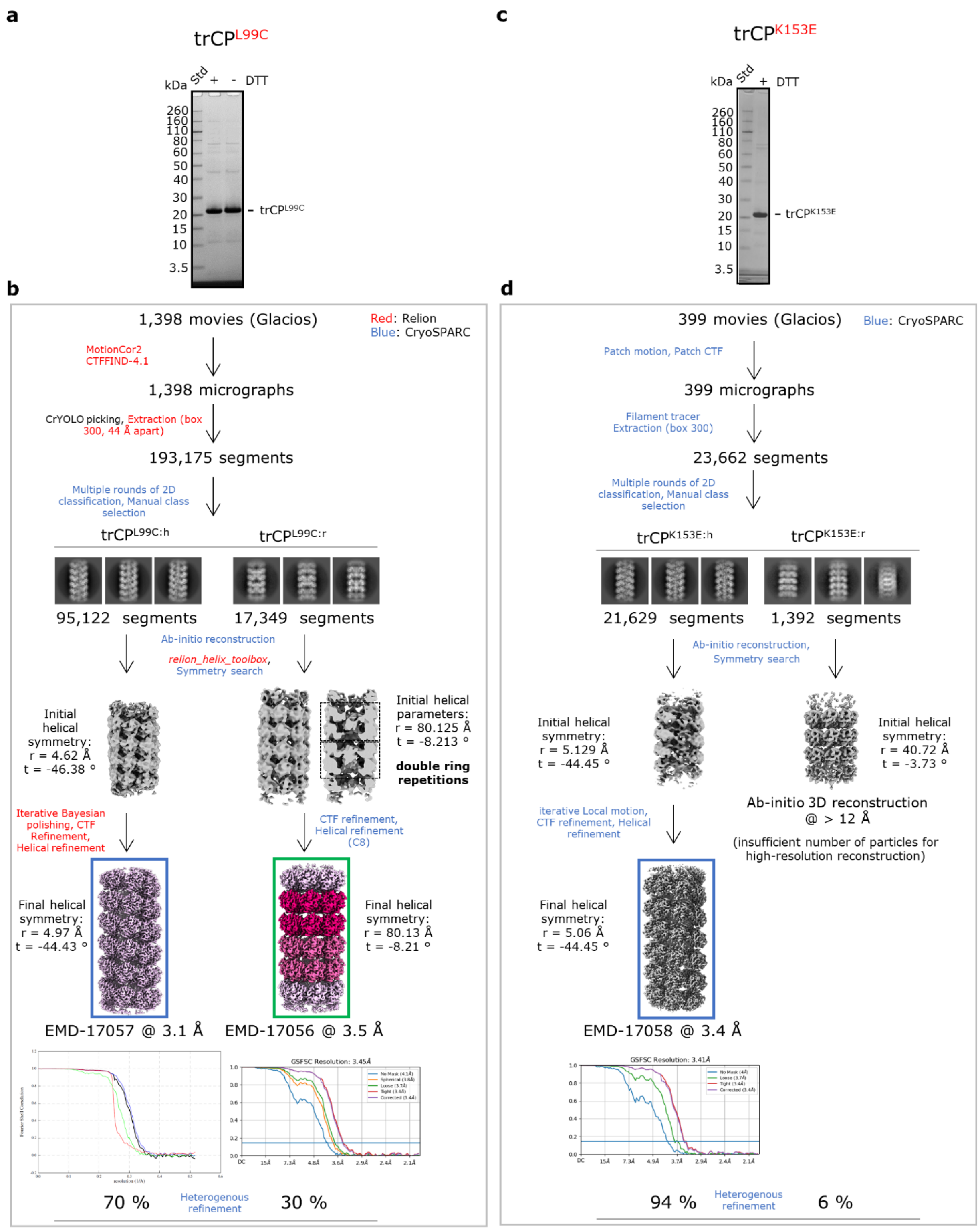

Supplementary Figure 8: Cryo-EM data processing for trCP^{L99C} and trCP^{K153E}.

a, SDS-PAGE analysis of trCP^{L99C} sample under reducing or oxidizing conditions (+/- DTT). **b,** Workflow of cryo-EM data processing for trCP^{L99C} assemblies. In the trCP$^{L99C:r}$ form, a distinct pattern of double-ring

repeats was observed in the 2D classes and the corresponding 3D reconstruction. **c,** SDS-PAGE analysis of trCP^{K153E} sample. **d,** Cryo-EM data processing workflow for trCP^{K153E} assemblies. Std: molecular weight standards. The source data for panels a and c are provided as Supplementary Data file.

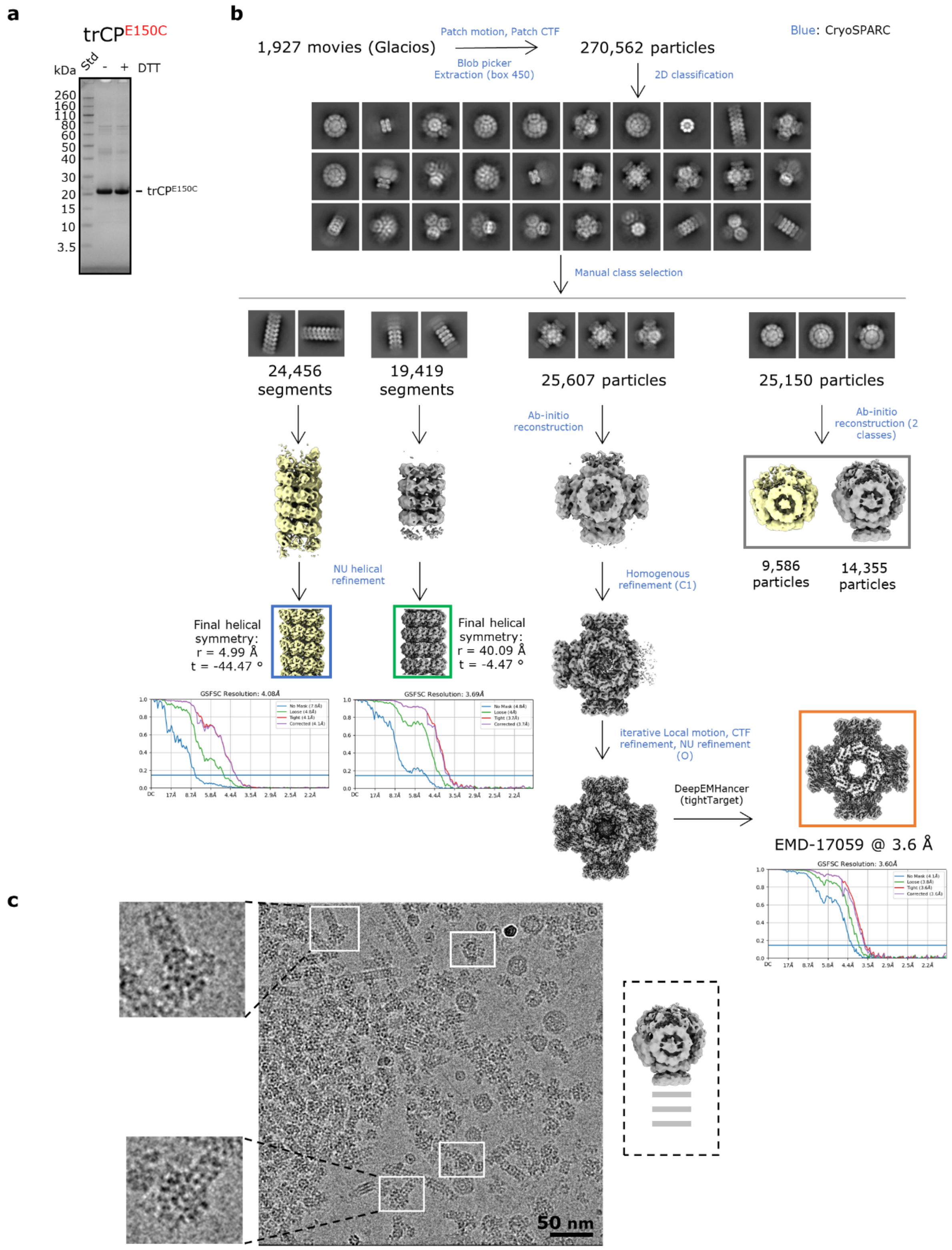

Supplementary Figure 9: Cryo-EM processing of the trCP^{E150C} sample.

a, SDS-PAGE analysis of trCP^{E150C} sample under reducing or oxidizing conditions (+/- DTT). Std: molecular weight standards. The source data is provided as Supplementary Data file. **b,** Workflow of cryo-

EM data processing for 4 different types of trCP^{E150C} assemblies. The final reconstructions of RNA-free helical (blue rectangle) and stacked-ring filaments (green rectangle) or cross-shaped (orange rectangle) and spherical junctions (dark gray rectangle) are marked **c,** Mapping of trCP^{E150C} spherical junctions (white rectangle) described in (b) on cryo-EM micrograph, with a scheme (right) indicating additional rings (gray lines) stacking on the central spherical body (3D reconstruction).

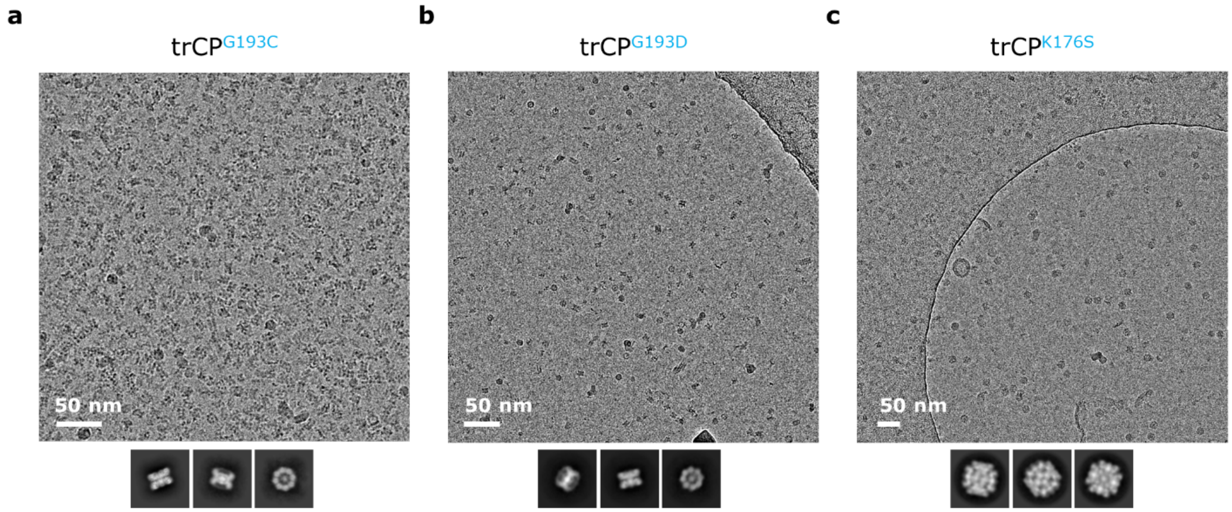

Supplementary Figure 10: Cryo-EM micrographs and 2D class averages of P-side mutants in addition to those shown in Fig. 4.

a-c, Representative micrographs and particle 2D class averages of **a,** trCP^{G193C}. **b,** trCP^{G193D}. **c,** trCP^{K176S}.

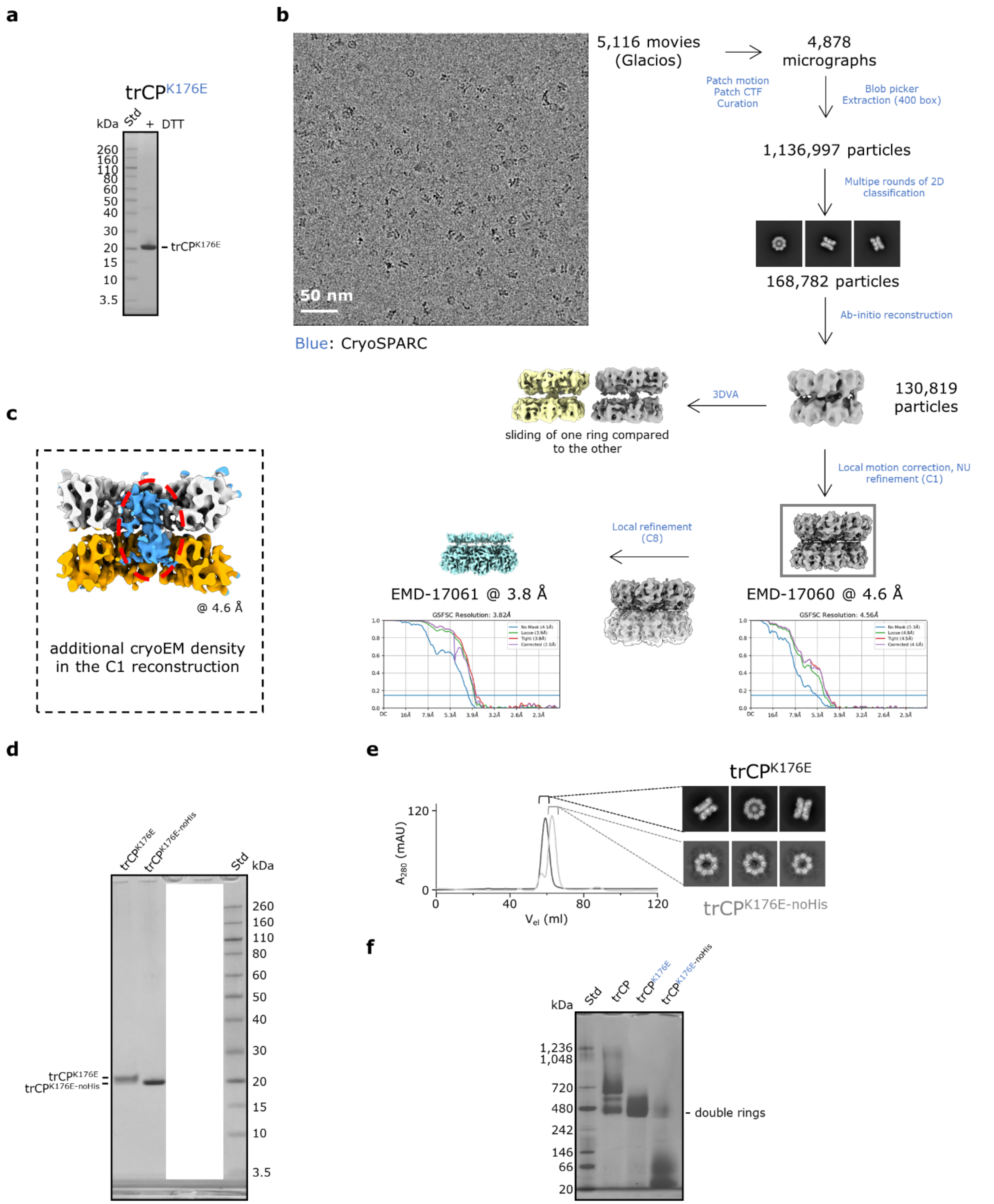

Supplementary Figure 11: Analysis of trCP^{K176E}.

a, SDS-PAGE analysis of trCP^{K176E}. **b,** Workflow of cryo-EM data processing for trCP^{K176E} H2H double-rings. Local refinement on one ring was performed due to the flexibility associated with shifting one ring relative to the other. **c,** Additional cryo-EM density, colored in blue, in the lumen (red dashed ellipse) of

the H2H double rings, assigned to C-terminal His_6-tag clustering. **d,** SDS-PAGE analysis of $trCP^{K176E}$ before and after removal of the His_6-tag ($trCP^{K176E\text{-}noHis}$). Other samples that do not belong to this panel were removed from the image (white filled rectangle). **e,** Comparison of SEC analysis of $trCP^{K176E}$ and $trCP^{K176E\text{-}noHis}$ with representative cryo-EM 2D class averages of the two samples. **f,** Native-PAGE analysis of trCP, $trCP^{K176E}$ and $trCP^{K176E\text{-}noHis}$. Std: molecular weight standards. The source data for panels a, d and f are provided as Supplementary Data file.

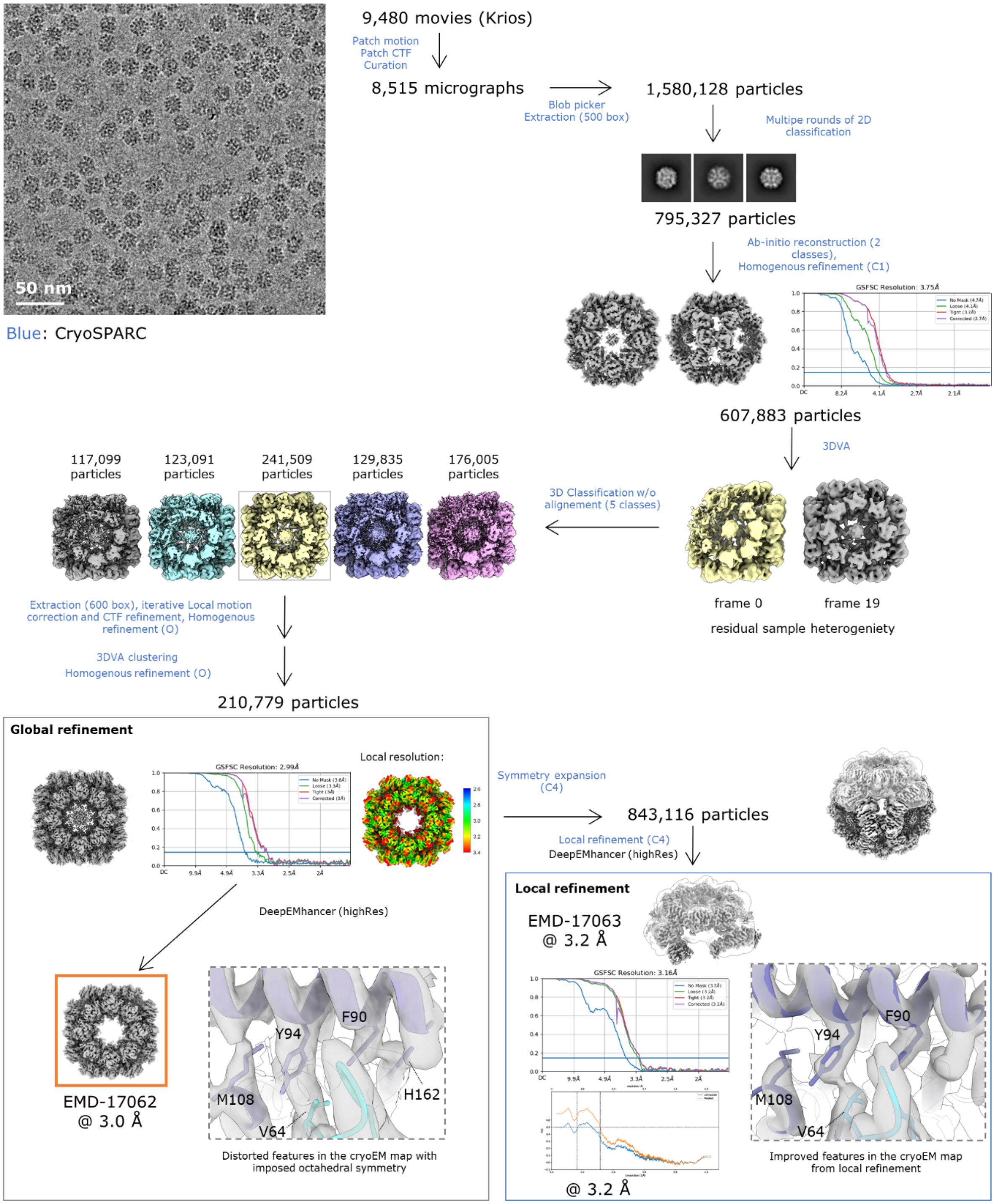

Supplementary Figure 12: Cryo-EM data processing for cubic trCP^{K176C} particles.

Workflow of cryo-EM data processing for trCP^{K176C} cubes. Globally refined map with imposed octahedral symmetry (O) showed distorted features, for example at positions V64, Y94, F90 and H162, shown in sticks (gray rectangle at the bottom left), which were improved using local refinement of one ring (blue rectangle, bottom right).

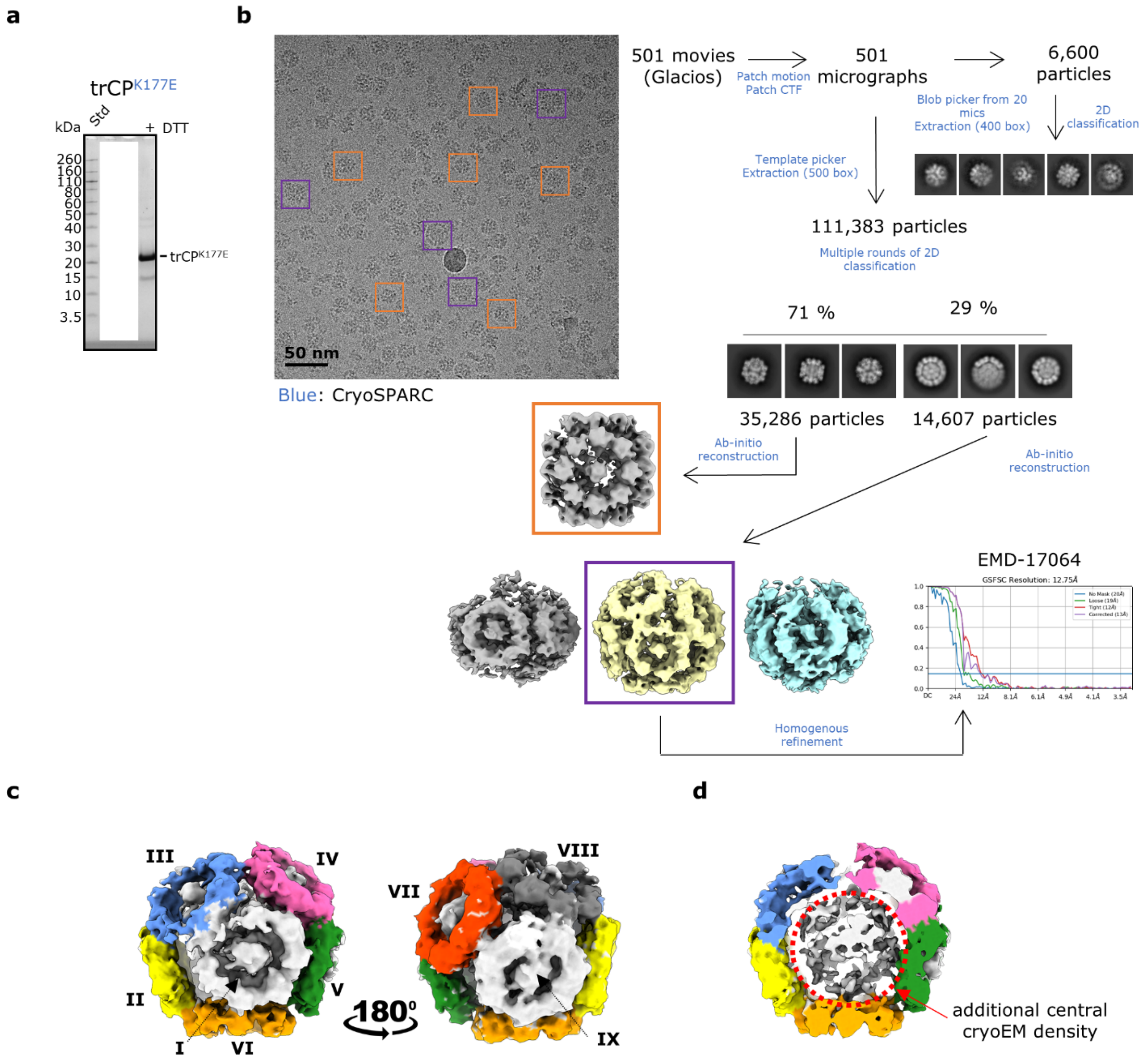

Supplementary Figure 13: Cryo-EM data processing for trCP^{K177E}.

a, SDS PAGE analysis of trCP^{K177E}. Other samples not belonging to this panel have been removed from the image (white filled rectangle). Std: molecular weight standards. The source data is provided as Supplementary Data file. **b,** Workflow of cryo-EM data processing for trCP^{K177E}. Boxes on micrograph indicate cube-shaped (orange) and spherical (purple) particles. **c,** Front and back views of Ab-initio 3D reconstruction of trCP^{K177E} spherical particle, which consists of 9 rings, each numbered and shown in a different color. **d,** A cross-sectional view of the spherical particle with additional cryo-EM density (red dotted circle) in the center, showing no distinct shape and thus uninterpretable. Front and back ring density has been removed for clarity.

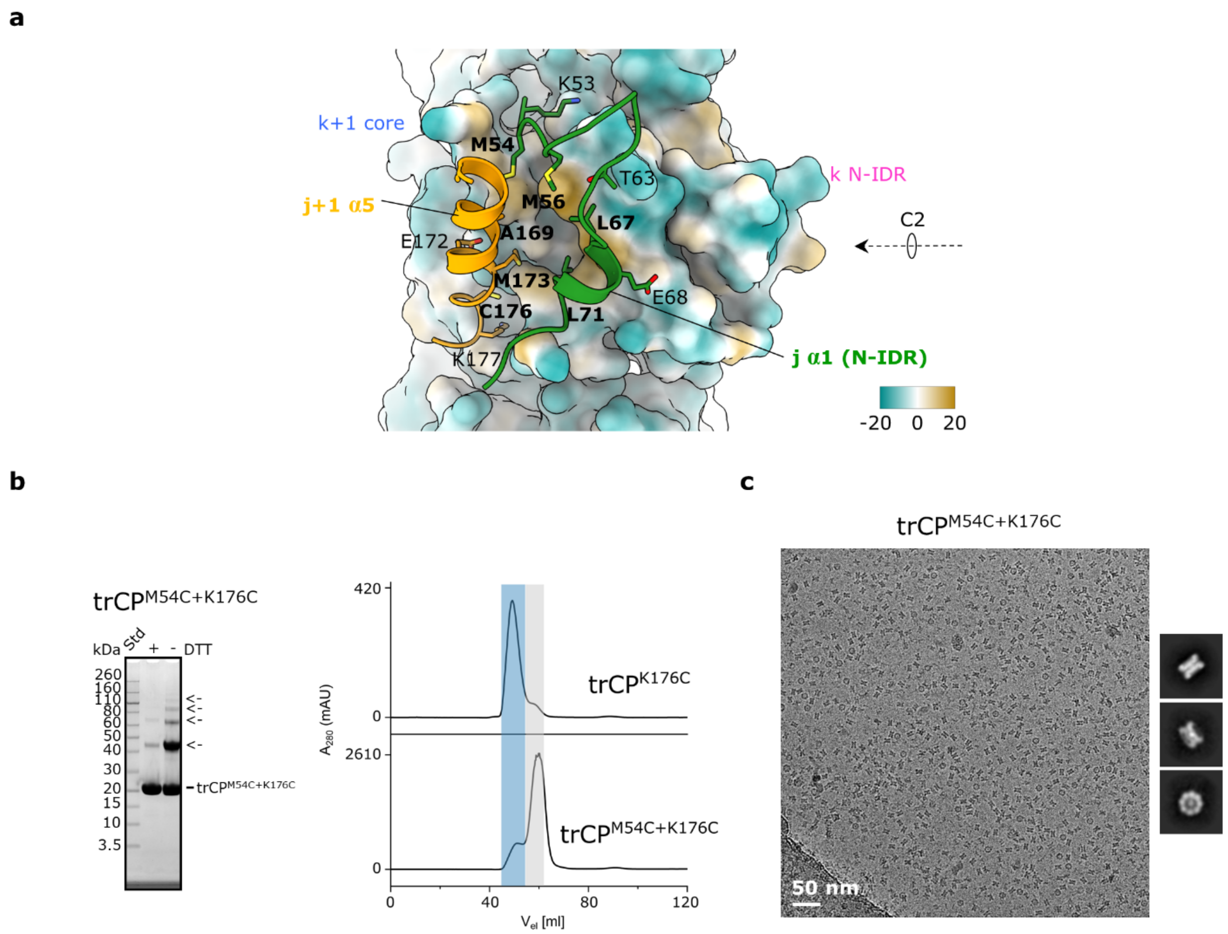

Supplementary Figure 14: The cubic trCP^{K176C} assembly is held together mainly by hydrophobic interactions.

a, Interaction interface between α1 and α5-helices from j and j+1 from one octameric ring (shown in green and yellow ribbon, respectively) bound to k and k+1 subunits (as also marked on Fig. 4a) from the adjacent ring, shown in surface representation with coloring based on lipophilicity potential (bottom right). The molecular lipophilicity potential was calculated using the standard Fauchere method[4] (e^{-d}) with lipophilicity potential increasing from -20 to +20. For clarity, only the residues involved in the interaction are shown. Interacting residues with hydrophobic side-chains are marked with bold letters. **b,** Left: SDS-PAGE analysis of trCP$^{M54C+K176C}$. The arrows indicate positions of trCP$^{M54C+K176C}$ oligomers. Right: Comparison of SEC analysis (HiLoad Superdex 200 16/600) of trCP^{K176C} and trCP$^{M54C+K176C}$. The source data is provided as Supplementary Data file. **c,** Representative cryo-EM micrograph and corresponding 2D class averages of trCP$^{M54C+K176C}$ H2H double rings.

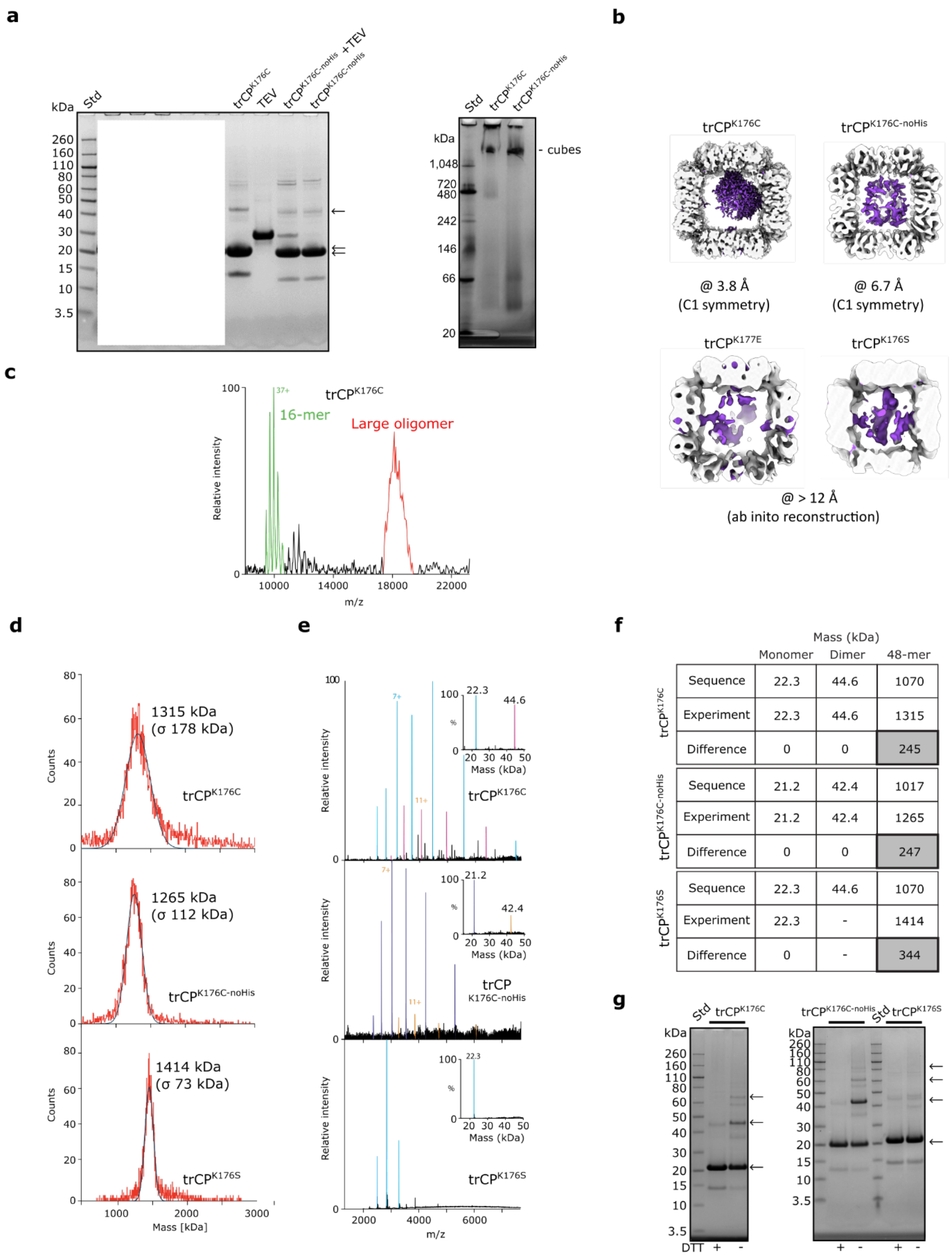

	Mass (kDa)		
	Monomer	Dimer	48-mer
**trCP^K176C**			
Sequence	22.3	44.6	1070
Experiment	22.3	44.6	1315
Difference	0	0	245
**trCP^K176C-noHis**			
Sequence	21.2	42.4	1017
Experiment	21.2	42.4	1265
Difference	0	0	247
**trCP^K176S**			
Sequence	22.3	44.6	1070
Experiment	22.3	-	1414
Difference	0	-	344

Supplementary Figure 15: Analysis of cubic particles composed of different trCP-derivatives.

a, SDS-PAGE (left) and native-PAGE (right) of trCP^{K176C} before and after TEV protease cleavage (trCP$^{K176C\text{-}noHis}$). trCP$^{K176C\text{-}noHis}$+TEV labels a sample before purification and trCP$^{K176C\text{-}noHis}$ after Ni-NTA chromatography removal of the His_6-tagged TEV protease. The bands corresponding to trCP^{K176C} or trCP$^{K176C\text{-}noHis}$ with their dimers are marked with arrows. Other SDS-PAGE samples that do not belong to this panel have been removed from the image (white filled rectangle). **b,** Comparison of the asymmetric cryo-EM reconstructions with their overall resolutions of various cubic assemblies showing an additional uninterpretable cryo-EM density in the center of the cube in purple. The front and back-side ring densities have been removed for clarity. **c,** Native mass spectrum of the trCP^{K176C} sample with charge states corresponding to 16-mer (green), and an unresolved species at high m/z ratios corresponding to large oligomers that cannot be mass measured, but could potentially be the cubic 48-mer (red). **d,** Mass photometry spectra of trCP^{K176C} (top), trCP$^{K176C\text{-}noHis}$ (middle) and trCP^{K176S} (bottom) with a symmetrical peak at a mass between 1.27 and 1.41 MDa, larger than expected 48-mer mass (1.07 MDa), consistent with entrapped cargo. **e,** Mass spectrometry data obtained under denaturing conditions for trCP^{K176C} (top), trCP$^{K176C\text{-}noHis}$ (middle) and trCP^{K176S} (bottom) with major charge series corresponding to monomer masses (cyan and purple) as well as minor charge series (pink and orange). **f,** Experimental masses from mass photometry and mass spectrometry (‘experiment’) compared to those calculated for 48-mer from the protein sequences (‘sequence’). The discrepancies observed between the experimental and expected mass of the complex indicate bound cargo within the cube. **g,** SDS-PAGE analysis of cubic particles in reducing and oxidizing conditions (+/- DTT). The bands corresponding to the mutant trCPs and their oligomers are indicated with arrows. Std: molecular weight standards. The source data for panels a and g are provided as Supplementary Data file.

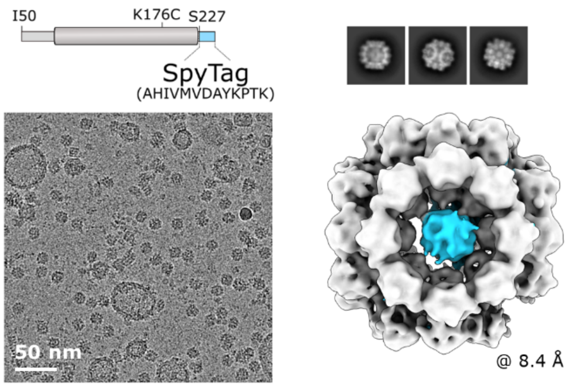

Supplementary Figure 16: Replacement of His_6-tag with SpyTag does not affect cubic formation.

Top left: schematic representation of construct with SpyTag (cyan) at the C-terminus of $trCP^{K176C}$. Bottom left: representative cryo-EM micrograph of $trCP^{K176C\text{-}SpyTag}$, 2D class averages (top right) and corresponding 3D reconstruction (bottom right) of assembled cubic particles. Additional density in the center of the octameric rings, corresponding to the SpyTag, is colored in cyan.

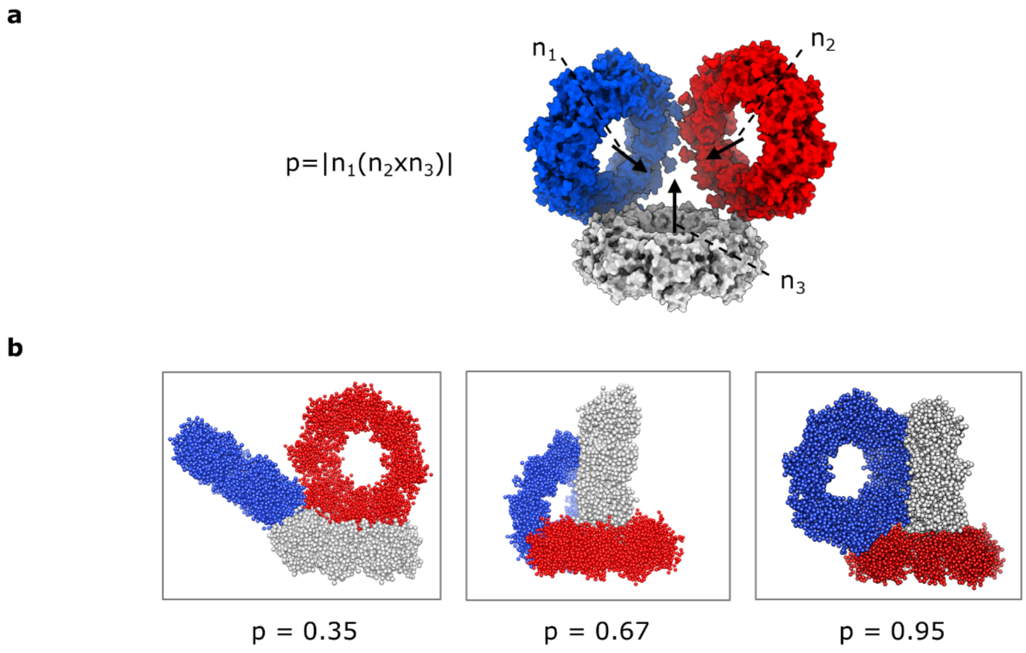

Supplementary Figure 17: Molecular dynamics (MD) simulations of the assembly of trCP^{K176C} rings. **a,** Representation of triple scalar product (p) revealing relative orientations of rings in triplets. When the mutual ring orientation is orthogonal, p has the value of 1. **b,** Snapshots of three octameric rings (triplets) with specific triple scalar product values, detected in the last 2 μs of the MD simulation. Values of the product were chosen based on the maxima of distribution for trCP^{noHis} (p=0.35) and trCP$^{K176C\text{-}noHis}$ (p=0.67 and p=0.95) on Fig. 5e.

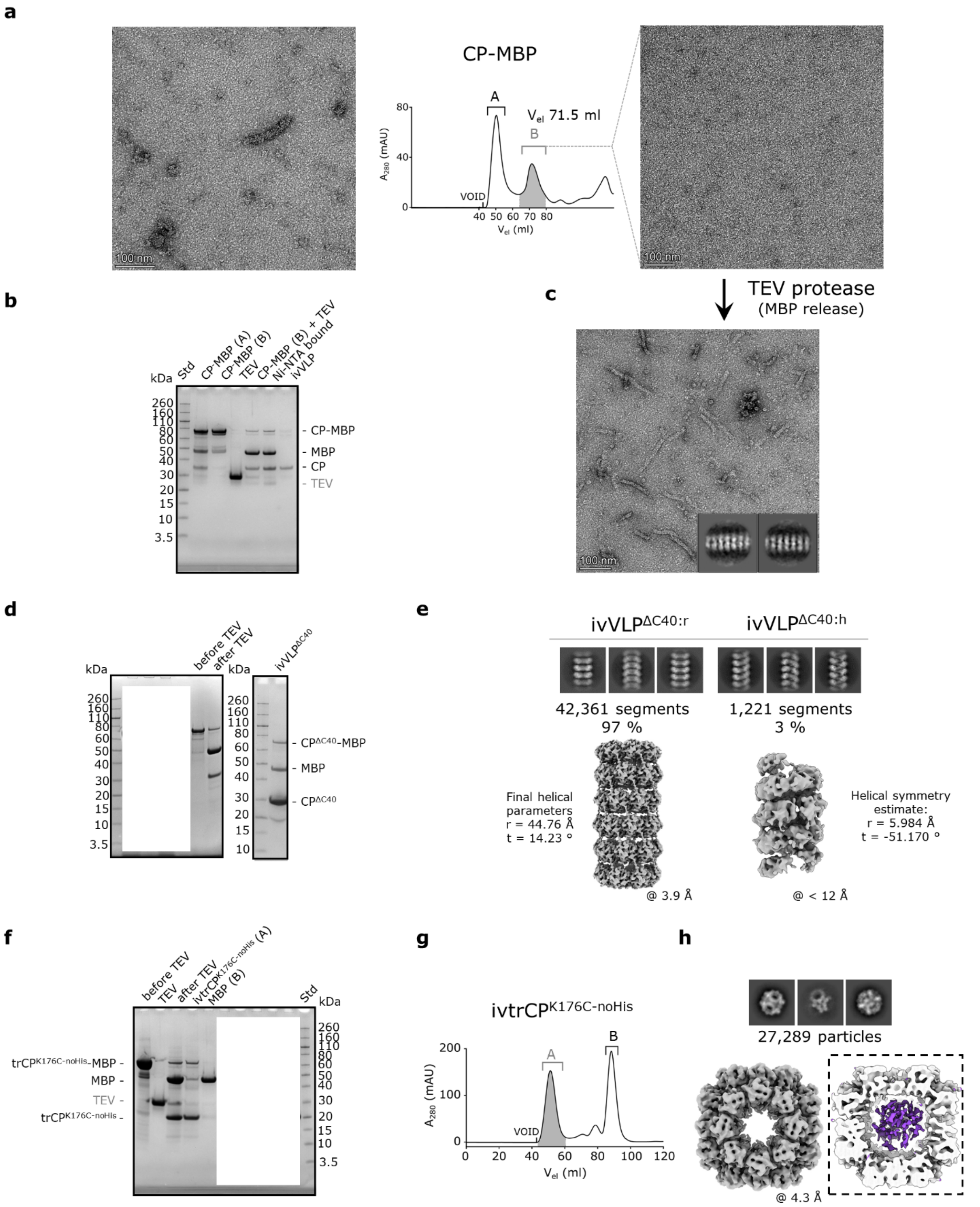

Supplementary Figure 18: ***In vitro*** **self-assembly of nanoparticles triggered by TEV-protease promoted release of CP derivatives from fusion with MBP.**

a, Left: nsTEM micrograph of CP-MBP sample. The sample was further purified via SEC (HiLoad Superdex 200 16/600) (middle) with elution profile showing proteins eluting in two major fractions (A and B). Both fractions contained CP-MBP fusion (panel b), but according to protein standards as reported by the column manufacturer (GE Healthcare) separated under the same conditions (PBS, 1 ml min^{-1}) (human albumin (66 kDa, V_{el} ~ 64 ml) and IgG (158 kDa, V_{el} ~ 75 ml)), the fraction B should contain CP-MBP monomer (75.3 kDa, V_{el} 71.5 ml). Fraction B was visualized by nsTEM (right). **b,** SDS-PAGE analysis of CP-MBP of SEC fractions 'A' and 'B' (middle panel a) and CP-MBP (fraction 'B') after TEV cleavage ('CP-MBP(B)+TEV'). 'Ni-NTA bound' is the fraction that remained on Ni-NTA column and 'ivVLP' is the fraction that was in the flow-through and contained *in vitro* assembled VLPs ('ivVLP'). **c,** nsTEM micrographs of the *in vitro* formed VLPs (ivVLP) after TEV cleavage from fraction B, with 2D class averages (inset) of the particles indicating the presence of stacked ring VLPs. **d,** Left: SDS-PAGE analysis of $CP^{\Delta C40}$-MBP sample before ('before TEV') and after incubation with TEV protease ('after TEV'). The sample mixture ('after TEV') was purified via the VLP purification procedure, as described in Methods. Right: SDS PAGE of the final sample of *in vitro* assembled nanotubes ('$ivVLP^{\Delta C40}$') after the VLP isolation procedure. Other samples not belonging to this panel have been removed from the image (white solid rectangle). **e,** Cryo-EM 2D class averages and 3D reconstructions of $ivVLP^{\Delta C40}$ forms with percentage of observed particle populations and (bottom) overall resolutions of corresponding 3D reconstructions in Å. **f-g**, SDS-PAGE analysis (f) of $trCP^{K176C\text{-}noHis}$-MBP before ('before TEV') and after incubation with TEV protease ('after TEV'). After TEV cleavage, the sample was further purified via SEC (g, HiLoad Superdex 200 16/600). In this process, $ivtrCP^{K176C\text{-}noHis}$ eluted around 50.3 ml (fraction A), which is characteristic of cubic $trCP^{K176C}$ particles, and the cleaved MBP eluted in fraction B. Other samples that do not belong to this panel have been removed from the image (white filled rectangle). **h,** Cryo-EM 2D class averages (top) and 3D reconstruction of the $ivtrCP^{K176C\text{-}noHis}$ cubic assemblies (left). The overall resolution is given in Å. Right: Asymmetric reconstruction of the cubic $ivtrCP^{K176C\text{-}noHis}$ assembly with additional central density in purple. Front- and back-side ring have been removed for clarity. Std: molecular weight standards. The source data for panels b, d and f are provided as Supplementary Data file.

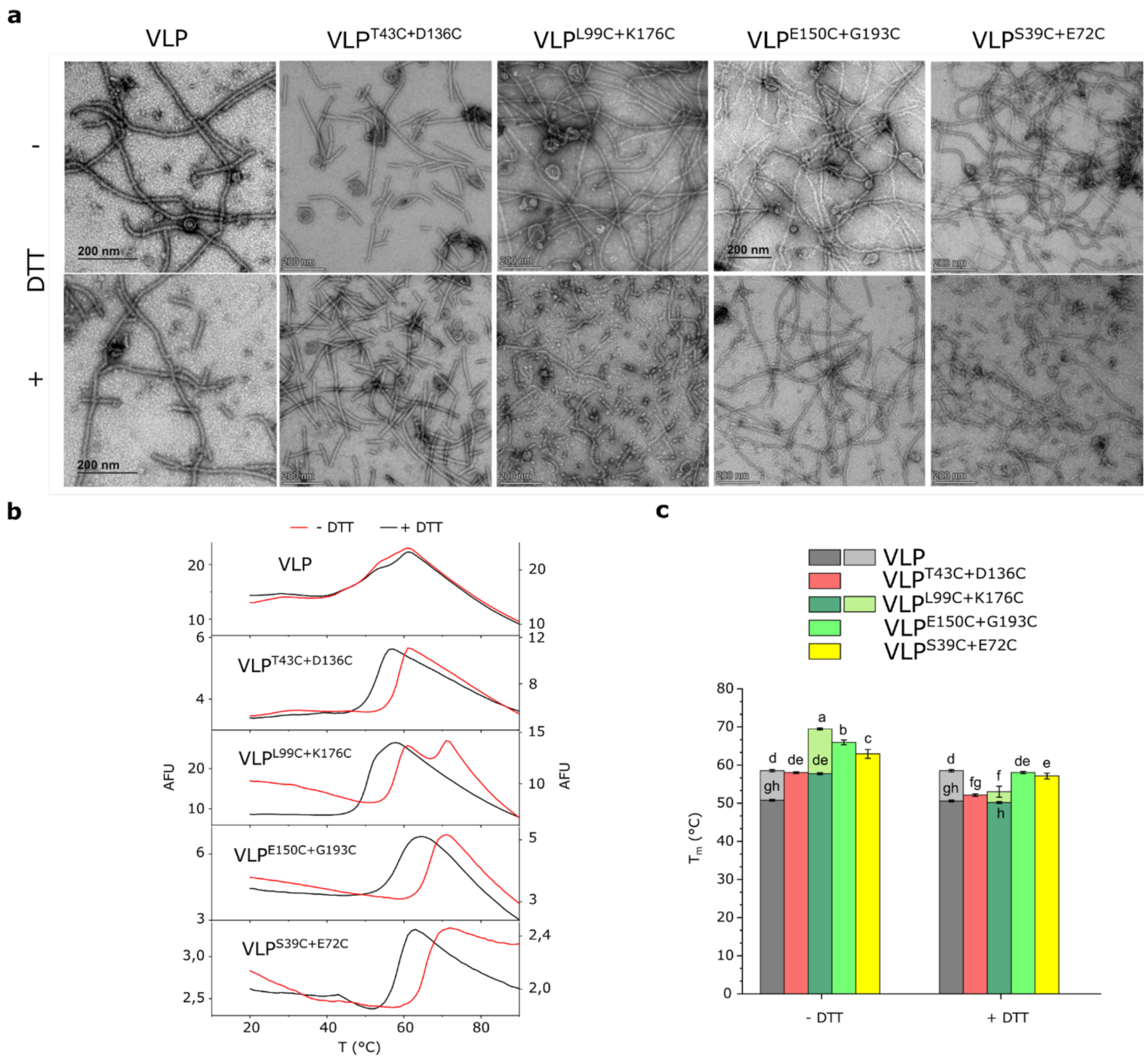

Supplementary Figure 19: Visualization of VLPs formed by CP double Cys mutants and their increased thermal stability.

a, nsTEM micrographs of VLPs formed by wild type CP or its double Cys mutants under reducing (+ 1 mM DTT) or oxidizing (- DTT) conditions in PBS, incubated for 10 min at 20 °C. **b**, Comparison of the thermal stability of double Cys mutants and wild-type CP measured by DSF. Raw data are shown on (b) and the respective T_m values on panel **c** as mean ± SD (*N* = 6). PBS buffer under reducing conditions (+DTT) or oxidizing conditions (- DTT) conditions. Statistical significance in (c) was determined by one-way ANOVA with Tukey's multiple comparison test (p=0.001). Multiple comparison was done performed between all measured T_m values, with labels a to h denoting a statistically significant difference. The source data for panels b and c are provided in the Supplementary Data file.

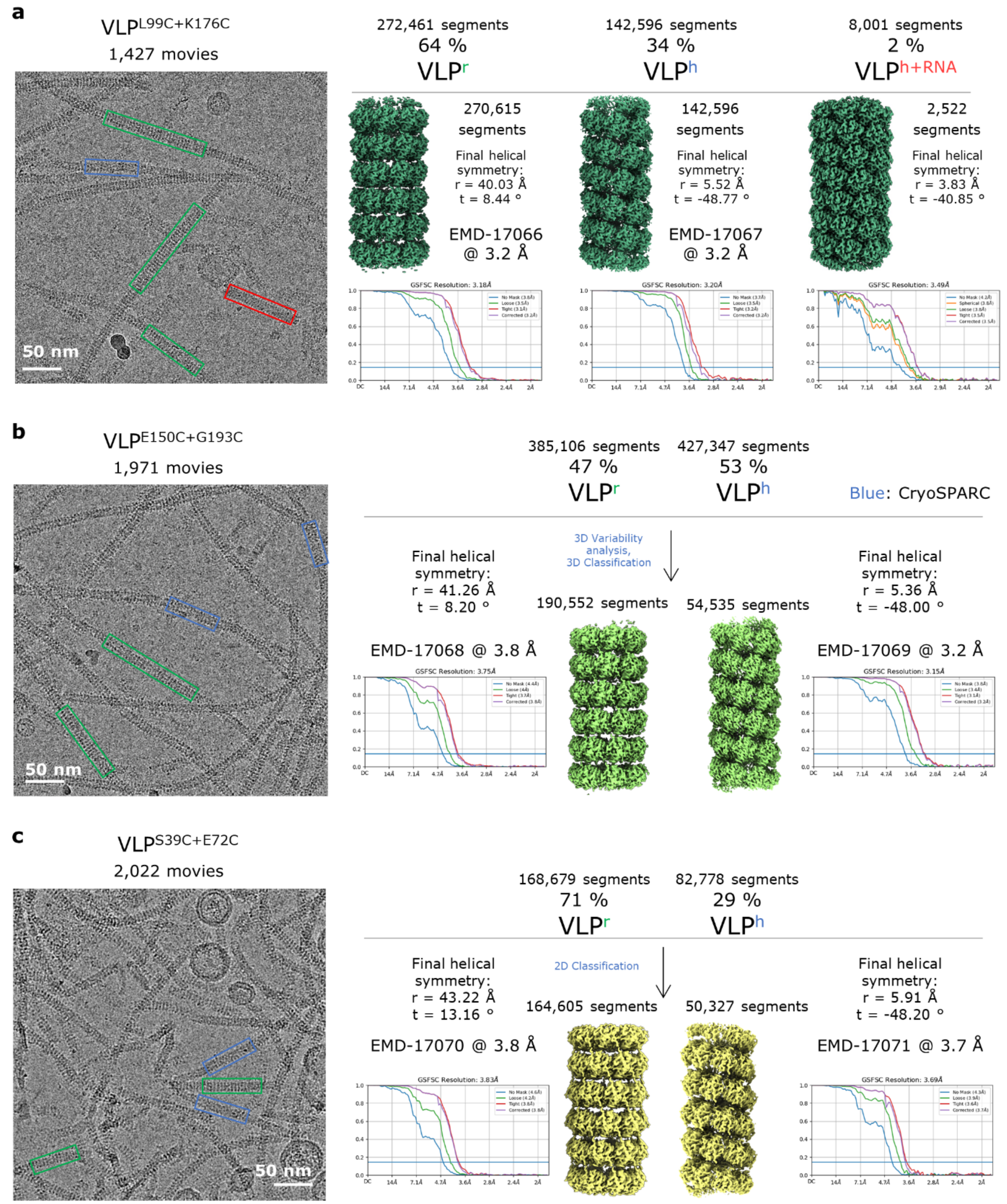

Supplementary Figure 20: Cryo-EM analysis of disulfide-bond stapled VLPs.

a, $VLP^{L99C+K176C}$ cryo-EM micrograph (left) with filaments of different architectures ($VLP^{L99C+K176C:r}$ green, $VLP^{L99C+K176C:h}$ blue, $VLP^{L99C+K176C:h+RNA}$ red), their relative proportions (in %) and corresponding 3D reconstructions (right). **b,** $VLP^{E150C+G193C}$ cryo-EM micrograph (left) showing mapped filaments of different architectures (stacked-ring in green, helical without RNA in blue), their relative proportions (in %) and

corresponding 3D reconstructions (right). **c,** $VLP^{S39C+E72C}$ cryo-EM micrograph (left) showing mapped filaments (stacked-ring in green, helical without RNA in blue) of different architectures, their relative proportions and corresponding 3D reconstructions (right).

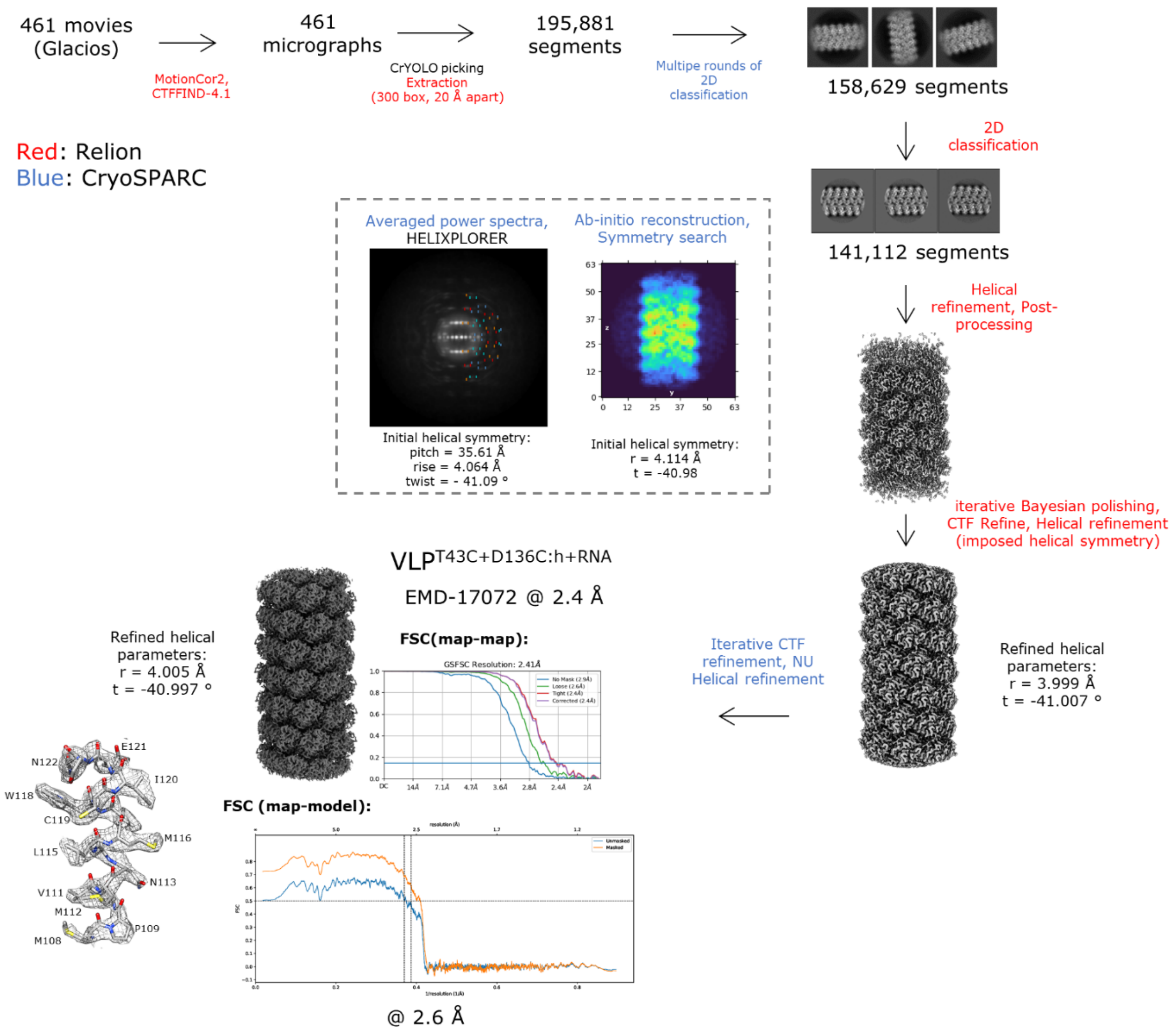

Supplementary Figure 21: Cryo-EM analysis of VLP$^{T43C+D136C}$.

Workflow of cryo-EM data processing for VLP$^{T43C+D136C}$.

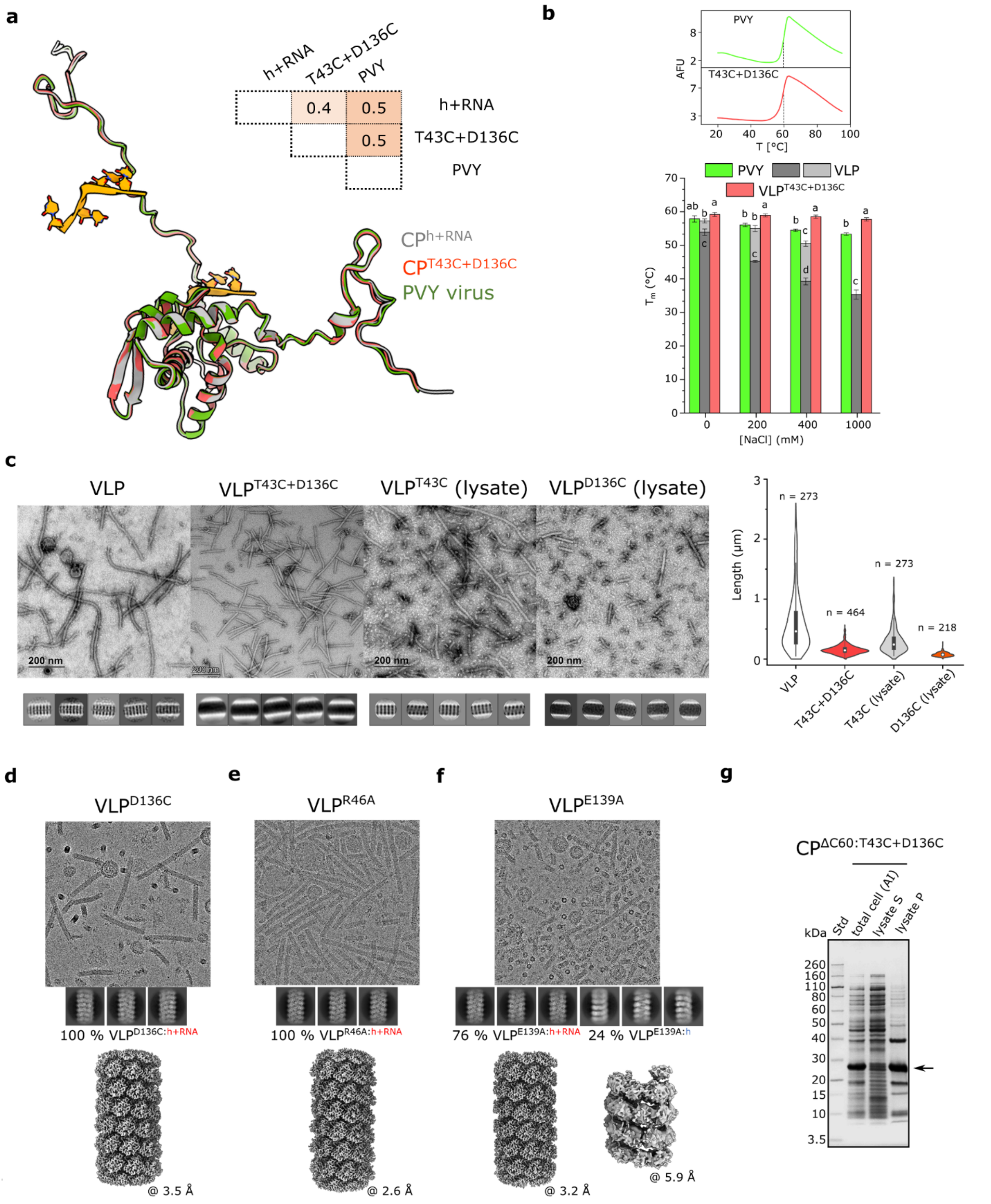

Supplementary Figure 22: Monomorphic VLP^{h+RNA} filaments are formed upon single amino acid replacement of the conserved residues D136/E139/R46.

a, Superposition of CP$^{T43C+D136C}$ (red), CP^{h+RNA} (gray) and CP^{PVY} from PVY virus (green, PDB ID 6HXX). Alignment was performed on all atoms, and RMSD values between all atom pairs are given in Å. **b,** Top:

representative DSF raw data for VLP$^{T43C+D136C}$ (red) and PVY virus (green) in 0.05 M SPG buffer pH 7.0 containing 0 mM NaCl, where the melting temperature (T_m) is indicated with a dashed line. Bottom: Comparison of T_m values from DSF measurement at different NaCl concentrations with T_m values shown as mean ± SD ($N = 6$). Statistical significance was determined by one-way ANOVA with Tukey's multiple comparisons test (p=0.001). Multiple comparisons were performed between measured T_m values within one condition (NaCl concentration), with labels a to d denoting statistically significant difference. **c,** Top left: nsTEM micrographs with corresponding nsTEM 2D class averages (bottom) for purified wild type VLPs and VLP$^{T43C+D136C}$, and for VLP^{T43C} and VLP^{D136C} directly from bacterial cell lysates. Right: violin plot of filament length distribution for wild type VLP, VLP$^{T43C+D136C}$, VLP^{T43C} and VLP^{D136C}. The 'n' above each violin corresponds to the number of measured filaments. The median value and the range above the 25th and below the 75th percentile are designated with a white circle and black solid rectangle, respectively. **d-f,** Cryo-EM micrograph, 2D class averages, and corresponding 3D reconstructions of the particles with their overall resolutions for VLP^{D136C} (d), VLP^{R46A} (e), and VLP^{E139A} (f). **g,** SDS PAGE analysis of CP$^{\Delta C60:T43C+D136C}$ sample after induction ('total cell (AI)'), soluble lysate ('lysate S') and pellet ('lysate P')). The arrow indicates the band corresponding to CP$^{\Delta C60:T43C+D136C}$, found predominantly in the pellet fraction. Std: molecular weight standards. The source data for panels b and c are provided as Supplementary Data file.

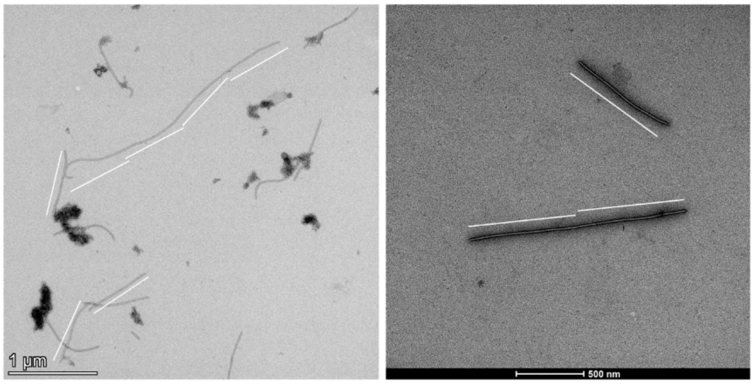

Supplementary Figure 23: Longitudinal merging of PVY virions.

Longitudinal association of PVY virions shown in two nsTEM micrographs. The length of the single PVY virion, marked with a white line on the micrographs, is approximately 740 nm.

a

RNA extraction

Sample	Starting VLP mass	$A_{260/280}$	Extracted RNA	$A_{260/280}$	$A_{260/230}$	Extracted RNA/ VLP mass
$VLP^{T43C+D136C}$	0.8607 mg	1.15	30.84 µg	2.22	2.52	35.83 µg mg^{-1} VLPs
$VLP^{\Delta C60}$	0.3984 mg	0.68	0.25 µg	1.91	1.03	0.63 µg mg^{-1} VLPs

b

RT-qPCR

	idnT Ct	mean *idnT* Ct	*CP* Ct	mean *CP* Ct
	26.3		7.0	
from VLPs_1	26.2	26.1 ± 0.2	6.9	7.0 ± 0.1
	25.9		7.0	
	28.2		8.1	
from VLPs_2	28.3	28.3 ± 0.1	8.1	8.0 ± 0.1
	28.3		7.9	

oligos

F_IdnT_qpcr	CTGTTTAGCGAAGAGGAGATGC
R_IdnT_qpcr	ACAAACGGCGGCGATAGC
F_CP_qpcr	ACGTGGTATGAAGCGGTACA
R_CP_qpcr	GCACCAAACCATAAGCCCAT

c

RNA sequencing

from VLPs_1		from VLPs_2		**from VLPs**
gene	% bp	gene	% bp	**mean % bp**
CP	70.27	*CP*	71.81	71.04
23S rRNA	0.19	23S rRNA	0.25	0.22
16S rRNA	0.12	16S rRNA	0.06	0.09
Other *E. coli* RNA	29.41	Other *E. coli* RNA	27.88	28.65
Sum	100.00	Sum	100.00	100.00
#Reads	5,187	#Reads	18,304	

d

mapping to 3' ends of coding sequences (CDS)

**from VLPs**	
CDS	mean TPM %
CP	74.91
hns	11.63
lpp	1.14
ompF	1.10
sodB	0.73
gdhA	0.59
raiA	0.47
tpx	0.36
rho	0.35
yhbY	0.29
Other genes	8.28
Sum	100.00

e

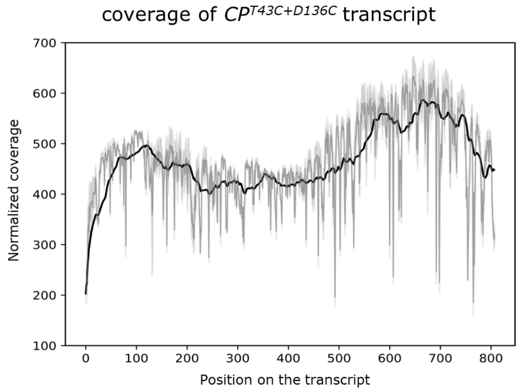

Supplementary Figure 24: RT-qPCR and nanopore sequencing of RNA extracted from $VLP^{T43C+D136C}$.

a, Amount of extracted RNA from filaments with encapsidated RNA ($VLP^{T43C+D136C}$). RNA-free filaments $VLP^{\Delta C60}$ were used as control. **b,** Reverse transcription-quantitative PCR (RT-qPCR) Ct values for RNA extracted from purified $VLP^{T43C+D136C}$, using *CP* and *idnT* qPCR primers. Data from two biological replicates ('from VLPs_1' and 'from VLPs_2') are shown. Three technical replicates were performed for each biological replicate. Bottom: oligonucleotides used in qPCR analysis. **c,** RNA nanopore sequencing. Table of the relative proportion (% bp) of total read bases that mapped to either to $CP^{T43C+D136C}$ coding sequence (*CP*), bacterial rRNA (23S and 16S rRNA) or other *E. coli* RNAs. The first two columns show the results of two biological replicates of RNA, extracted from VLPs ('from VLPs_1' and 'from VLPs_2'), with the respective percentage means in the third column. Values are presented as bp % values calculated by adding gene-specific base pairs (bps) and comparing them to the sum of all mapped bps. The total number of reads in each RNA nanopore experiment is indicated below with '#Reads'. **d,** Normalized coverage plot of $CP^{T43C+D136C}$ coding sequences (CDS) of RNA extracted from VLPs. The black and dark gray lines represent the smoothened and raw mean of normalized per base coverage of the two biological

replicates, respectively. The standard deviation between the two replicates is indicated with light gray. **e,** Table of the mean relative percentage of the number of transcripts for the ten most abundant transcripts, mapping to $CP^{T43C+D136C}$ or *E. coli* coding sequences. Values are presented in the form of adjusted transcripts per million (TPM) values (in %) and were determined after mapping reads to coding sequences using a special 3'-filtering that selected only reads that mapped near the 3' ends of CDS, i.e. near the stop codon (Methods).

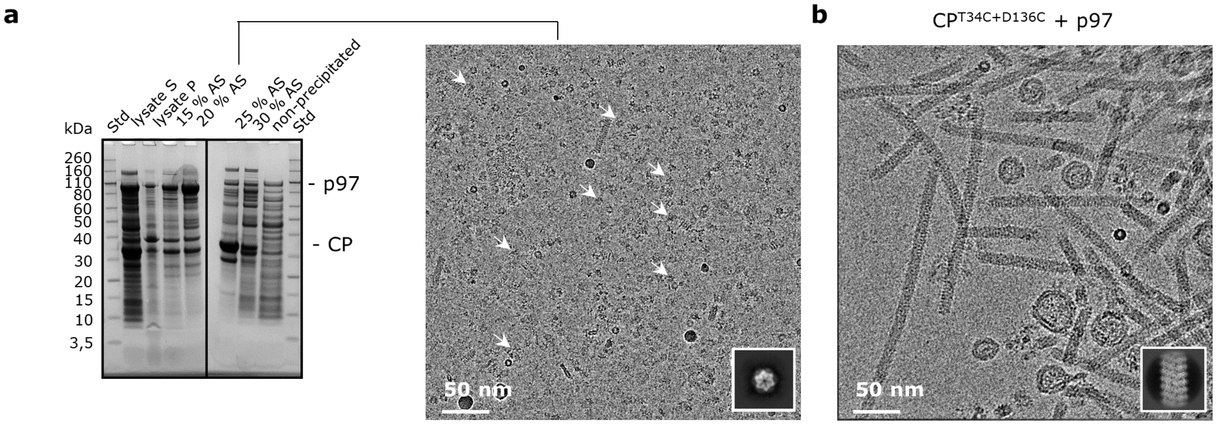

c

RNA sequencing

total cell_1		total cell_2		**total cell**
gene	% bp	gene	% bp	**mean** % bp
23S rRNA	56.49	23S rRNA	63.66	60.08
16S rRNA	30.58	16S rRNA	27.60	29.09
p97	7.54	*p97*	4.56	6.05
CP	3.65	*CP*	2.68	3.17
Other *E. coli* RNA	1.74	Other *E. coli* RNA	1.50	1.62
Sum	100.00	Sum	100.00	100.00
#Reads	41,181	#Reads	36,319	

from VLPs_1		from VLPs_2		**from VLPs**
gene	% bp	gene	% bp	**mean** % bp
CP	44.41	*p97*	39.61	40.99
p97	34.67	*CP*	37.56	37.14
23S rRNA	0.13	23S rRNA	0.32	0.23
16S rRNA	ND	16S rRNA	0.07	0.04
Other *E. coli* RNA	20.79	Other *E. coli* RNA	22.44	21.61
Sum	100.00	Sum	100.00	100.00
#Reads	3,086	#Reads	9,140	

ND... below detection limit

d

mapping to 3′ ends of coding sequences (CDS)

**total cell**		**from VLPs**	
CDS	mean TPM %	CDS	mean TPM %
CP	68.16	*CP*	71.21
p97	19.75	*hns*	12.16
hns	3.67	*lpp*	3.98
lpp	1.06	*sodB*	1.82
sodB	0.70	*p97*	1.81
raiA	0.68	*tpx*	0.94
ompF	0.67	*yhbY*	0.85
tpx	0.38	*raiA*	0.82
lacI	0.34	*ompF*	0.82
yhbY	0.33	*crl*	0.73
Other genes	4.26	Other genes	4.86
Sum	100.00	Sum	100.00

Supplementary Fig. 25: Analysis of protein and RNA samples obtained in co-expression experiment of CP$^{T43C+D136C}$ and p97.

a, Left: Two SDS-PAGE gels of samples obtained during precipitation with ammonium sulphate (AS) as the first step of VLP isolation, where percentage indicates the concentration of AS required for precipitation of this fraction. ‘lysate S’ and ‘lysate P’ are the soluble and insoluble fraction, obtained after cell lysis and centrifugation, while the ‘non-precipitated’ fraction contains proteins, that could not be precipitated in 30 % (*w/v*) AS. p97 and CP are enriched in different fractions (‘20 % AS’ and ‘25 % AS’, respectively). Right: cryo-EM micrograph of p97 hexamers (white arrows) in a particular fraction (‘20 % AS’), with a corresponding 2D class average of the particle in the lower right corner. The source data are provided as Supplementary Data file. **b,** Cryo-EM micrograph of purified VLPs, recovered from the ‘25 % AS’ fraction (A) and further purified via the VLP isolation procedure (Methods), with a corresponding 2D class average of the VLPs in the lower right corner. **c,** Tables of the relative fraction (% bp) of total read bases that mapped to either to *CP*$^{T43C+D136C}$ (CP) or *p97* (p97) coding sequence, bacterial rRNA (23S and 16S rRNA), or other *E. coli* RNAs, for total cell RNA (left; two biological replicates: ‘total cell_1’ and ‘total cell_2’) and for RNA, extracted from VLPs (right; two biological replicates: ‘from VLPs_1’ and ‘from VLPs_2’). In both cases, the first two columns represent the results for two biological replicates of RNA sample, with the respective percentage means in the third column. Values are presented as bp % values calculated by

adding gene-specific base pairs (bps) and comparing them to the sum of all mapped bps. The total number of reads in each RNA nanopore experiment is indicated below with '#Reads'. **d,** Table of the mean relative percentage of the number of transcripts for the top ten transcripts mapped to $CP^{T43C+D136C}$, *p97* or *E. coli* coding sequences. Values are presented in the form of adjusted transcripts per million (TPM) values (in percent) and were determined after mapping reads to coding sequences using a special 3'-filtering that selected only reads that mapped near the 3' ends of CDS, i.e. near the stop codon (Methods).

Supplementary Table 1. Cryo-EM map statistics for reconstructed filamentous VLPs supplemental to Table 1

Sample	**PVY virus**[a]	**VLP$^{L99C+K176C}$**			**VLP$^{E150C+G193C}$**		**VLP$^{S39C+E72C}$**		**VLP^{D136C}**	**VLP^{R46A}**	**VLP^{E139A}**		**ivVLP$^{\Delta C40}$**	
VLP architecture*	h+RNA	r	h	h+RNA	r	h	r	h	h+RNA	h+RNA	h+RNA	h	r	h
Percentage of particles detected in the sample (%)	100	64	34	2	47	53	71	29	100	100	76	24	97	3
**Symmetry**														
Point group	C1	C1	C1	C1	C1	C1	C1	C1	C1	C1	C1	C1	C8	C1
Helical rise (Å)	3.95	40.03	5.52	3.83	41.26	5.36	43.22	5.91	4.08	4.12	4.09	5.91	44.76	5.98
Helical twist (°)	-40.95	8.44	-48.77	-40.85	8.20	-48.00	13.16	-48.20	-40.96	-41.06	-41.01	-48.04	14.23	-51.17
**Map resolution (Å)**														
Map:Map FSC (Å, 0.143)	3.4	3.2	3.2	3.5	3.8	3.2	3.8	3.7	3.5	2.6	3.2	5.9	3.9	>12
**Deposition ID**														
EMDB (Map)	EMD-0297	EMD-17066	EMD-17067	/	EMD-17068	EMD-17069	EMD-17070	EMD-17071	EMD-17073	EMD-17074	EMD-17075	/	/	/

'*': r…stacked-ring; h+RNA…helical with RNA; h…helical without RNA; iv…*in vitro* produced. [a] data for PVY virus were obtained from [2].

Supplementary Table 2. Cryo-EM map statistics for reconstructed trCP assemblies supplemental to Table 1

Mutation	**No mutation**			**L99C**		**K153E**		**E150C**				**K176E**		**K177E**		**K176C -noHis**	**K176C (iv)**
Morphology*	H2T double rings	h	r	h	r	h	r	h	r	cj	sj	H2H double rings (global)	H2H double rings (local)	s	s	c	c
Percentage of particles detected in the sample (%)	93	2	1	70	30	94	6	26	21	27	26	100		29	71	100	100
**Symmetry**																	
Point group	C8	C1	C8	C1	C8	C1	C8	C1	C8	O	C1	C1	C8	C1	C1	O	O
Helical rise (Å)	39.30	4.90	39.53	4.97	80.13	5.06	40.72	4.99	40.09	N/A	N/A	N/A	N/A	N/A	N/A	N/A	N/A
Helical twist (°)	-3.59	-44.41	-3.59	-44.43	-8.21	-44.45	-3.73	-44.47	-4.47	N/A	N/A	N/A	N/A	N/A	N/A	N/A	N/A
**Map resolution (Å)**																	
Map:Map FSC (Å, 0.143)	2.9	3.5	3.7	3.1	3.5	3.4	>12	4.1	3.7	3.6	>12	4.6	3.8	12.8	>12	4.2	4.3
**Deposition ID**																	
EMDB (Map)	EMD-17053	EMD-17054	EMD-17055	EMD-17057	EMD-17056	EMD-17058	/	/	/	EMD-17059	/	EMD-17060	EMD-17061	EMD-17064	/	EMD-17065	/

trCP: truncated CP ($CP^{\Delta N49C40\text{-}CHis}$); '*': r…stacked-ring filaments; h+RNA…helical filaments with RNA; h…helical filaments without RNA. iv…*in vitro* produced; cj…cross-shaped junctions; sj…spherical junctions; s…spherical particles; c…cubic particles; N/A: not applicable.

Supplementary References

1. Feng, C. Q. *et al.* ITerm-PseKNC: A sequence-based tool for predicting bacterial transcriptional terminators. *Bioinformatics* **35**, 1469–1477 (2019).
2. Kežar, A. *et al.* Structural basis for the multitasking nature of the potato virus Y coat protein. *Sci Adv* **5**, eaaw3808 (2019).
3. Grech-Baran, M. *et al.* The Rysto immune receptor recognises a broadly conserved feature of potyviral coat proteins. *New Phytologist* **235**, 1179–1195 (2022).
4. M. Laguerre, M. Saux, J. P. Dubost, A. Carpy, MLPP: A Program for the Calculation of Molecular Lipophilicity Potential in Proteins. *Pharmacy and Pharmacology Communications*. **3**, 217–222 (1997).
