# Supplementary material for: From structural polymorphism to structural metamorphosis of the coat protein of flexuous filamentous potato virus Y
Source: Commun Chem. 2024 Jan 17;7:14. doi: 10.1038/s42004-024-01100-x (PMC10794713; doi:10.1038/s42004-024-01100-x)
Supplement: Supplementary file 3 — Description of Additional Supplementary Files [file 42004_2024_1100_MOESM3_ESM.pdf]

# Description of Additional Supplementary Files

**File name:** Supplementary Data 1

**Description:** Compiled source data for the main and supplemental figures, as described in the main article and supplementary information.
